# Development and Validation of Diagnostic KASP Markers for Brown Planthopper Resistance in Rice

**DOI:** 10.3389/fgene.2022.914131

**Published:** 2022-07-08

**Authors:** V. G. Ishwarya Lakshmi, M. Sreedhar, V. JhansiLakshmi, C. Gireesh, Santosha Rathod, Rajaguru Bohar, Santosh Deshpande, R. Laavanya, K. N. S. Usha Kiranmayee, Sreedhar Siddi, S. Vanisri

**Affiliations:** ^1^ Department of Genetics and Plant Breeding, College of Agriculture, Professor Jayashankar Telangana State Agricultural University (PJTSAU), Hyderabad, India; ^2^ Administrative Office, PJTSAU, Hyderabad, India; ^3^ ICAR-Indian Institute of Rice Research (IIRR), Hyderabad, India; ^4^ CGIAR Excellence in Breeding (EiB), CIMMYT-ICRISAT, Hyderabad, India; ^5^ International Crops Research Institute for the Semi-Arid Tropics (ICRISAT), Patancheru, India; ^6^ Agricultural Research Station, PJTSAU, Peddapalli, India; ^7^ Institute of Biotechnology, PJTSAU, Hyderabad, India

**Keywords:** rice, brown planthopper, SNP, GWAS, GBS, KASP

## Abstract

Rice (*Oryza sativa* L.) is an important source of nutrition for the world’s burgeoning population that often faces yield loss due to infestation by the brown planthopper (BPH, *Nilaparvata lugens* (Stål)). The development of rice cultivars with BPH resistance is one of the crucial precedences in rice breeding programs. Recent progress in high-throughput SNP-based genotyping technology has made it possible to develop markers linked to the BPH more quickly than ever before. With this view, a genome-wide association study was undertaken for deriving marker-trait associations with BPH damage scores and SNPs from genotyping-by-sequencing data of 391 multi-parent advanced generation inter-cross (MAGIC) lines. A total of 23 significant SNPs involved in stress resistance pathways were selected from a general linear model along with 31 SNPs reported from a FarmCPU model in previous studies. Of these 54 SNPs, 20 were selected in such a way to cover 13 stress-related genes. Kompetitive allele-specific PCR (KASP) assays were designed for the 20 selected SNPs and were subsequently used in validating the genotypes that were identified, six SNPs, viz, snpOS00912, snpOS00915, snpOS00922, snpOS00923, snpOS00927, and snpOS00929 as efficient in distinguishing the genotypes into BPH-resistant and susceptible clusters. *Bph17* and *Bph32* genes that are highly effective against the biotype 4 of the BPH have been validated by gene specific SNPs with favorable alleles in M201, M272, M344, RathuHeenati, and RathuHeenati accession. These identified genotypes could be useful as donors for transferring BPH resistance into popular varieties with marker-assisted selection using these diagnostic SNPs. The resistant lines and the significant SNPs unearthed from our study can be useful in developing BPH-resistant varieties after validating them in biparental populations with the potential usefulness of SNPs as causal markers.

## Introduction

Insect pests are important causal factors for major yield losses in rice crops and are detrimental to food security worldwide. Among the various insect pests, the brown planthopper (BPH; *Nilaparvata lugens* Stål) is extremely notorious and destructive that has advanced from non-significant to a prominent state and can cause up to 80% yield losses in rice (Rashid et al., 2017; Balachiranjeevi et al., 2019) with curtailment in the photosynthetic rate, nitrogen content, chlorophyll pigment, leaf area, and dry matter accumulation in most susceptible rice cultivars (Cagampang et al., 1974). Apart from serving as a carrier for ragged stunt and grassy stunt diseases (Jena and Kim, 2010; Sarao et al., 2016), the BPH causes hopperburn symptoms (Backus et al., 2005) which leads to major yield losses in farmer fields. Four different biotypes of the BPH that vary in virulence against different genotypes have been reported in rice ecologies (Khush et al., 1985; Brar et al., 2009). Of these, biotype 4 is exclusive to the Indian subcontinent (Liu et al*.,* 2015; Ren et al., 2016; Prahalada et al., 2017).

Conventional measures to reduce BPH damage with the application of chemical insecticides are not only costly but also environmentally hazardous, polluting the ecosystem and disrupting the natural balance of BPH predators such as mirid bugs viz.*, Cyrtorhinus lividipennis* (Reuter) and *Tytthus parviceps* (Reuter) that hold the pest population (JhansiLakshmi et al., 2010a and b). For building a sustainable pest management strategy, the correct combination of breeding for resistance and control measures that must be established to diminish the BPH’s ecological fitness while maintaining the pest below economic threshold levels was suggested by Bosque-Perez and Buddenhagen (1992). Of the different strategies, the use of resistant varieties as a measure of host plant resistance (HPR) has been considered among the most economic and potent methods to protect against virulent BPH populations to achieve long-term and broad-spectrum resistance (Khush, 2001). As the vast majority of BPH-resistant genes do not confer broad-spectrum resistance to different biotypes of the BPH, the production of new rice cultivars with a broad range of resistance to BPH populations derived from diverse genetic sources is the need of the hour. With the constant efforts of rice scientists since the 1960s, around 42 BPH resistance genes and more than 70 quantitative trait loci (QTLs) have been identified and assigned to different rice chromosomes (Akanksha et al.*,* 2019; Balachiranjeevi et al., 2019; Muduli et al., 2021; Tan et al., 2021) but only few genes, mostly *Bph17*, *Bph3/32*, *Bph31*, and *Bph33(t)* showed broad-spectrum resistance to Indian biotype 4 (Liu et al*.,* 2015; Ren et al., 2016; Prahalada et al., 2017; Naik et al., 2018).

Over the past few decades, crop breeding has been altered to a great extent and taken into a positive direction with cutting-edge contemporary genomic technologies (Bohar et al., 2020; Ahmar et al., 2021). Genomics-assisted breeding led to the development of several crop varieties in rice (Fukuoka et al., 2010), groundnut (Varshney et al., 2013), chickpea (Varshney et al., 2013), pigeon pea (Varshney et al., 2013; Bohra and Kumar Varshney, 2020), wheat (De et al., 2009), and maize (Xu et al., 2009; Leng et al., 2017) with the introduction of molecular approaches in varietal development. Recent breakthroughs in next-generation sequencing (NGS) technology and high-throughput SNP marker-based genotyping technologies have become the unprecedented tools for the accelerated identification of markers linked to desirable traits in the field of plant breeding (Peng et al., 2020). Since their discovery, SNPs have become the most promising tool for the incorporation of desired genes because of their wide dispersal within genomes and suitability for high-throughput automated genotyping (Tamura et al., 2014; Yang et al., 2019). With several re-sequencing projects, QTL mapping, gene mapping, and GWAS in rice provide abundant information about an enormous number of genetic loci and SNPs that accord to BPH resistance. However, trait-linked SNPs need to be perfectly validated to use them as diagnostic markers in future marker-assisted breeding (MAB) programs. The identified SNPs through different approaches can be converted to KASP assays and validated for the linked traits (Gouda et al., 2021).

Kompetitive allele-specific PCR (KASP) assay is one of the most widely used uniplex genotyping platforms which is simple, fast, and economical. The method permits the detection of SNPs with high precision and has potential usefulness in MAS related to many breeding programs (He et al., 2014; Yang et al., 2019). KASP-based genotyping services are offered through several private and public service providers. For instance, the International Crops Research Institute for the Semi-Arid Tropics (ICRISAT) has led the High-Throughput Genotyping Project (HTPG) for SNP genotyping using KASP in collegiality with International Rice Research Institute (IRRI), International Maize and Wheat Improvement Center (CIMMYT), and the CGIAR EiB (Excellence in Breeding) platform with the financial support of the Bill & Melinda Gates Foundation. The HTPG facilitated shared industrial-scale low-cost, high-throughput SNP genotyping for CGIAR, NARS, and small–medium private sector organizations, mainly for forward breeding applications through collaboration, knowledge allocation, and new technologies adoption in 18 crops including rice (Bohar et al.*,* 2020). Presently, the HTPG has transitioned into an EIB platform, and the shared services are offered as EiB low-density genotyping service (EiB-LDSG) (CGIAR, 2021). KASP-based genotyping was well used through the HTPG/EiB-LDSG with the successful development of markers for target traits along with the use of the existing KASP markers for introgression of target traits in a number of crops including groundnut (Parmar et al., 2021), sorghum (Mwamahonje et al., 2021), potato (Kante et al., 2021; Sood et al., 2022), cassava (Thuy et al., 2021), and rice (Arbelaez et al., 2019). Among the marker panels of rice with the HTPG, few SNPs related to BPH resistance for genes *Bph17*, *Bph32*, and *Bph9* are available (Excellence in breeding, 2021) and can be genotyped for further use as diagnostic markers.

Authors have performed a genome-wide SNP discovery using GBS data of MAGIC *indica* population and identified SNPs associated with BPH resistance. To validate the identified SNPs through the KASP assay using HTPG services, the present study was undertaken with an aim to confirm the presence of *Bph17*, *Bph32*, and *Bph9* genes in the germplasm and to validate the newly identified SNPs to use them as diagnostic markers in institutional MAB programs.

## Materials and Methods

### Plant Materials Used for Genome-Wide SNP Discovery

The panel comprised 391 MAGIC *indica* lines (Supplementary Table S1) that were produced at IRRI, Philippines, by inter-crossing eight elite *indica* founder parents through 8-way F_1_ inter-crossing (Bandillo et al., 2013; Satturu et al., 2020). The BPH damage score and GBS data of these lines were used for genome-wide association studies. Serial numbers for the 391 lines were given from M1 to M395 and four lines were not included as the GBS data were missing.

### Plant Materials Used for Validation

The validation panel comprised 83 rice genotypes comprising 30 BPH gene differentials, 36 MAGIC lines, 11 3K genome lines, six germplasm lines (including popular varieties and landraces), three resistant (PTB33, RathuHeenati, RP 2068-18-3-5), and two susceptible checks (TN1 and BPT5204) (Table 1). The materials were acquired from the Institute of Biotechnology, PJTSAU, Hyderabad, and ICAR-Indian Institute of Rice Research (IIRR), Hyderabad.

**TABLE 1:** List of 83 genotypes and checks used in the study.

S. No.	Genotype	S. No.	Genotype	S. No.	Genotype
	BPH gene Differentials		MAGIC lines		MAGIC lines
1	Mudgo	31	M1	61	M312
2	IR 64	32	M4	62	M344
3	ASD 7	33	M61	63	M359
4	Milyang 63	34	M80	64	M362
5	RathuHeenati	35	M88	65	M364
6	Babawee	36	M123	66	M384
7	ARC 10550	37	M131		Germplasm lines
8	Swarnalatha	38	M179	67	IET23993
9	T12	39	M182	68	BM71
10	Chinsaba	40	M187	69	RPV1355
11	Pokkali	41	M189	70	KNM118
12	IR 65482-7-216	42	M190	71	10-3
13	IR 71033-121–15	43	M192	72	Telangana Sona
14	MUT NS1	44	M201		3K Genome Lines
15	OM 4498	45	M227	73	3K-19
16	RP 2068-18-3-5	46	M229	74	3K-47
17	MO1	47	M240	75	3K-53
18	MTU 1010	48	M262	76	3K-59
19	RP BIO 4918-230S	49	M267	77	3K-132
20	IR 26	50	M272	78	3K-168
21	IR 40	51	M276	79	3K-187
22	IR 66	52	M278	80	3K-200
23	IR 72	53	M279	81	3K-202
24	Utrirajappan	54	M284	82	3K-290
25	Ndiang Marie	55	M286	83	3K-322
26	Sinna Sivappu	56	M289		Checks
27	Balamwee	57	M293		PTB33
28	IR 62	58	M296		RathuHeenati
29	RathuHeenati accession	59	M304		RP 2068-18-3-5
30	IR 65482-136-2-2	60	M306		TN1, BPT5204

### Brown Planthopper Insects and Mass Rearing

BPH adults were collected from the rice fields of ICAR-IIRR, Rajendranagar, Hyderabad, and the pure colonies were reared and managed at a temperature of 25 ± 5°C with a relative humidity of 70 ± 5% on 60 days old potted plants of the susceptible variety (TN1) under glasshouse conditions. Cages of 70 × 62 × 75 cm dimensions were mounted on wooden benches with glass-paneled doors on one side and a wire mesh on all the other sides for mass rearing. This was followed by the collection of adult gravid female hoppers and their release on pre-cleaned potted plants of TN1. The process was repeated after 3–4 days of egg-laying and the hatched nymphs were used for screening when they had attained the appropriate age.

### Evaluation of Rice Genotypes for BPH Response

**FIGURE 1:**
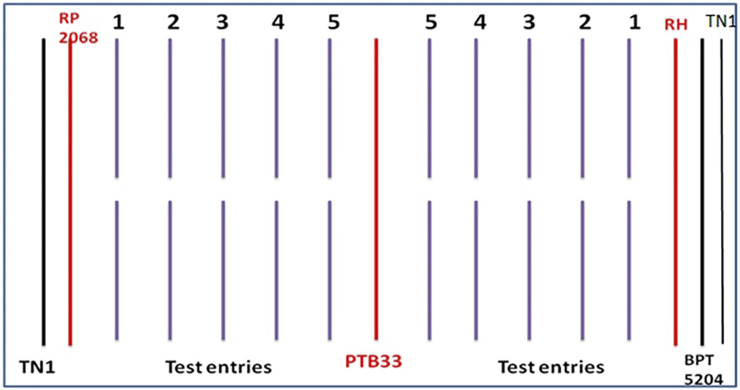
Layout of entries along with resistant and susceptible checks evaluated for BPH resistance using SSST method (RH = RathuHeenati).

The standard seedbox screening technique (SSST) (Heinrichs et al., 1985) was used to assess the extent of BPH resistance across the 83 rice genotypes at the seedling stage. Seeds of the 83 genotypes were pre-soaked and sown in rows of 60 × 45 × 10 cm in seed boxes accommodating 20– 25 seedlings per row in augmented block design conducted during *Kharif* 2018 and *Rabi* 2018–19 (Figure 1) at the ICAR-Indian Institute of Rice Research, Entomology glasshouse. Four screening trays (blocks) comprising 20 test entries each and three entries in the fifth tray were evaluated with the checks being replicated in all trays at random. Twelve days after sowing, first-instar nymphs were delivered on the seedlings at 6–8 nymphs/seedling. The tray was turned 180° when TN1 plants on one side showed symptoms to have even reactions on both sides. The Standard Evaluation System (SES) for rice (Standard Evaluation System for Rice, 2013) was used to rate the damage of the test lines (Table 2) when 90% of the TN1 plants were killed. The plants with scores 0–1.0 were considered as highly resistant, 1.1–3.0 as resistant, 3.1– 5.0 as moderately resistant, 5.1– 7.0 as moderately susceptible, 7.1– 8.9 as susceptible, and 9.0 as highly susceptible.

**TABLE 2:** Classification of resistance based on the damage reaction (Standard Evaluation System for Rice, 2013).

Resistance score	Plant state	Rating
0	No damage	Highly resistant
1	Very slight damage	
3	Lower leaf wilted with two green upper leaves	Resistant
5	Two lower leaves wilted with one green upper leaf	Moderately resistant
7	All three leaves wilted but stem still green	Moderately susceptible
9	All plants dead	Susceptible

### Genotyping-by-Sequencing (GBS)

The genotyping-by-sequencing (GBS) method was followed for genotyping 391 MAGIC lines through Illumina HiSeq (Elshire et al., 2011). The raw genotyping data of 391 MAGIC lines (Raghavan et al., 2017) were retrieved (SNPs and Phenotypes Data, 2021) and processed through the TASSEL GBS pipeline to obtain polymorphic SNPs. These SNPs were curated by the GBS pipeline using TASSEL 3.0.169 software (Glaubitz et al., 2014) based on <30% missing, locus homozygosity, and minor allele frequency (MAF) of 0.05.

### Genome-Wide Association Studies (GWAS) and Candidate Genes

During 2016 and 2017, 391 MAGIC *indica* lines were screened for resistance against the BPH using the SSST at the ICAR-Indian Institute of Rice Research and IBT, PJTSAU, Hyderabad, by Satturu et al. (2020). The GWAS analysis was conducted using the FarmCPU model that identified 31 annotated significant SNPs associated with 13 stress-related genes (Satturu et al., 2020).

For our study, the average BPH score values of all the 391 MAGIC *indica* lines previously evaluated by Satturu et al. (2020) were considered for calculating the best linear unbiased predictions (BLUPs). The cured polymorphic SNPs obtained from GBS and across-year phenotypic data were engaged for conducting the GWAS through R software-based GAPIT (Genetic Association and Prediction Integrated Tools) (Lipka et al., 2012) analysis package for identifying marker-trait associations. The MAGIC lines were treated as unrelated individuals in the GWAS since the MAGIC population has an insignificant population structure (Bossa-Castro et al., 2018; Satturu et al., 2020). To account for the relatedness among accessions of the panel, the kinship matrix was established using the Centered IBS method. Three single-locus models such as the general linear model (GLM) (Price et al., 2006), mixed linear model (MLM) (Zhang et al., 2005), and Settlement of MLM Under Progressively Exclusive Relationship (SUPER) (Wang et al., 2014) and three multi-locus models viz., multi-locus mixed-model (MLMM) (Segura et al., 2012), Fixed and Random Model Circulating Probability Unification (FarmCPU) (Zhang et al., 2010), and Bayesian-information and Linkage-disequilibrium Iteratively Nested Keyway (BLINK) (Huang et al., 2019) were engaged to derive the marker-trait associations. According to the general distribution of all *p*-values of the SNPs for BPH resistance, a suggestive significance threshold of *p*-values < 0.001 was considered. The results from all the models were studied to find out the consistency and repeatability of the associations. Based on the deviation of the observed statistic values from the expected statistic values in Q-Q plots, the significant SNPs identified through the GLM model were selected for further annotation studies. To spot the candidate genes underlying the haplotypes of interest, rice genome browsers including the Rice Genome Annotation Project-Michigan State University Rice Genome Annotation Project database (Osa1) Release 7 were searched and 23 SNPs involved in stress resistance pathways were collected using SNPEff 4.3T software (SnpEff and SnpSift, 2021). Of these 23 SNPs, 20 SNPs along with the previously identified 31 SNPs from the FarmCPU model (Satturu et al., 2020) were selected in such a way to cover all the 13 stress-related genes for designing KASP assays in our validation studies.

### DNA Extraction for Uniplex SNP Validation

For validation studies, leaf samples of the 83 rice genotypes along with checks raised at the College farm, PJTSAU, Rajendranagar, Hyderabad, were collected after 21 days of planting. Two leaf discs of 4 mm diameter were collected from each genotype using the paper punching machine and placed into a 12 x 8-well strip tube of 96-well microtiter plate provided by Intertek-Agritech laboratory, Hyderabad (Intertek AgriTech, 2021), by taking enough care to avoid DNA contamination and for robust genotypic data quality. The leaf discs were then oven-dried at 45°C for 12 h to remove moisture and stored at room temperature. These leaf discs were subsequently used for genomic DNA isolation, quantification, and genotyping. DNA isolation was carried out using LGC oKtopure™ automated high-throughput sbeadex™ (surface-coated superparamagnetic beads) DNA extraction and purification system (Biosearch technologies, 2021b) and the steps were followed according to the manufacturer’s instructions at AgriTech-Intertek Pvt. Ltd., Hyderabad, India. The leaf samples were homogenized by steel bead grinding in 96-deep-well plates and an extraction buffer in the sbeadex™ kit (Biosearch technologies, 2021b) was added. Purification of the extracted DNA was performed using sbeadex™ coated super paramagnetic particles with a surface chemistry that catches nucleic acids from a sample. The purified DNA was eluted and used for quantification and genotyping experiments. The genotyping data were generated using the KASP assay.

### Genotyping Using KASP Assay

KASP genotyping was carried out using the HTPG services provided at Intertek, Hyderabad, through the EiB platform funded by the CGIAR and Bill & Melinda Gates Foundation (Bohar et al*.*, 2020). To develop KASP genotyping assays for SNPs related to BPH resistance, flanking sequences of the selected 20 SNPs were retrieved using SAMtools1.1 software (Li et al., 2009) by aligning the genomic sequences with chromosome positions to the reference genome. A minimum of 100 bases of sequences for each of the identified SNPs having 50 bases on either flanking side was extracted. These sequences were further used to design KASP primers/assays (LGC, Biosearch Technologies) and were used for validating the genotypes used in the study by using the HTPG platform (Supplementary Table S2). Along with these 20 SNPs, SNPs specific to *Bph17*, *Bph32*, and *Bph9* (Table 3) available with HTPG services (Excellence in breeding, 2021) were used for validating the rice panel as these are stable resistance genes providing valuable defense against a BPH attack.

**TABLE 3:** List of reported functional SNPs validated in the study.

S. No.	Gene	SNP	Chromosome	Positive allele	Negative allele	Position (Mb)
1	*Bph17*	17-1	4	T	C	6.9
2	*Bph17*	17-2	4	G	C	6.9
3	*Bph17*	17-3	4	G	A	6.9
4	*Bph32*	32	6	G	C	1.2
5	*Bph9*	9-2	12	A	C	22.8

### SNPviewer Software

The cluster plots generated from the SNP genotype datasets were graphically viewed in SNPviewer v.4.1.2 (Biosearch technologies, 2021a). The HTPG genotyping result files present in a CSV (comma-separated value) format were given as input for grouping the allele calls to differentiate the resistant and susceptible genotypes based on the presence of favorable SNP alleles. Accordingly, homozygotes and heterozygotes for the SNP loci were distinguished based on the difference in fluorescence using the SNPviewer software.

## Results

### Analysis of Variance for BPH Resistance

**FIGURE 2:**
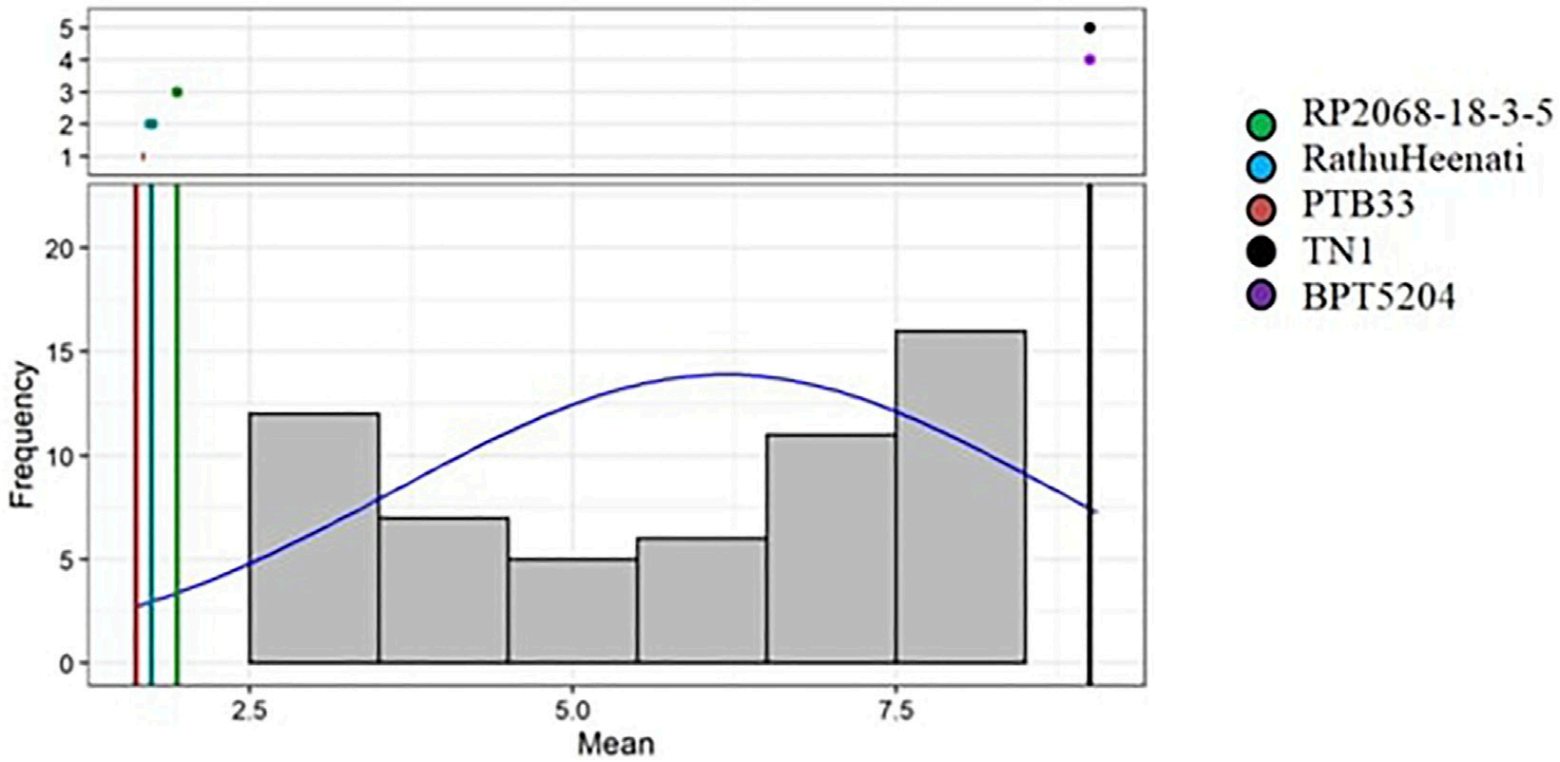
Frequency distribution of adjusted mean BPH scoring values of the rice genotypes.

The phenotypic reaction of 83 rice genotypes and checks evaluated using SSST exhibited varied levels of resistance response (Supplementary Table S3). The analysis of variance for damage scores revealed significant differences among the genotypes for various sources of variation as shown in Table 4. The block effect (unadjusted), treatment effects (adjusted and unadjusted), and effects due to checks, varieties, and checks versus varieties were all significant, whereas the adjusted block effects were non-significant. On comparing the damage scores of both the seasons, 13 of the 83 entries were resistant, 15 were moderately resistant, 12 genotypes were moderately susceptible, 34 were susceptible, and the remaining nine genotypes were found to be highly susceptible. The 13 resistant genotypes (Supplementary Figure S1, Table 5) included nine MAGIC lines (M4, M88, M179, M182, M192, M229, M240, M312, and M344), three gene differentials (RP 2068-18-3-5, RP Bio4918-230S, and RathuHeenati), and a landrace (10-3) with a low damage score varying between 1.3 and 3.0. Considerable skewness was observed for the genotypes more toward susceptibility with most of the genotypes showing damage scores between 7.1 and 8.9 as depicted by frequency distribution-based adjusted mean values (Figure 2).

**TABLE 4:** Analysis of variance for brown planthopper scoring of 83 rice genotypes and checks in augmented RCBD.

Source of variation	d.f	Mean sum of squares (MSS)				
		*Kharif* 2018	*Kharif* 2018	*Rabi* 2018–19	*Rabi* 2018–19	Overall
		(Trial I)	(Trial II)	(Trial I)	(Trial II)	
Block (ignoring treatments)	4	2.23 **	2.72 **	2.45 **	2.96 **	1.41 **
Treatment (eliminating blocks)	87	9.56 **	9.99 **	9.39 **	10.18 **	9.52 **
Checks + var vs. var	83	6.24 **	6.63 **	6.08 **	6.81 **	6.2 **
Block (eliminating check + var.) (adj)	4	0.01	0.02	0.01	0.02	0.01
Entries (ignoring blocks) (adj)	87	9.67 **	10.11 **	9.5 **	10.31 **	9.59 **
Checks	4	79.56 **	78.15 **	80.14 **	78.35 **	78.55 **
Varieties	82	6.38 **	5.53 **	6.43 **	5.69 **	5.82 **
Checks vs. varieties	1	38.34 **	60.59 **	49.24 **	53.8 **	49.89 **
Residuals	16	0.01	0.01	0.01	0.01	0.0037

d.f: degrees of freedom ** Significance at 0.5% probability level.

**TABLE 5:** Grouping of genotypes based on the reaction to BPH damage.

Damage score	Reaction	Genotypes
1.1–3.0	Resistant	M4, M88, M179, M182, M192, M229, M240, M312, M344, 10-3, RP Bio4918-230S, **RathuHeenati, RP 2068–18-3-5, and PTB33 (checks)**
3.1–5.0	Moderately resistant	M61, M187, M201, M276, M284, M286, M359, BM71, IET23993, RPV1355, Mudgo, Swarnalatha, T12, Sinna Sivappu, and RathuHeenati accession
5.1–7.0	Moderately susceptible	M80, M278, M293, M289, M279, M296, M384, IR64, OM4498, IR72, 3K-202, and 3K-59
7.1–8.9	Susceptible	KNM118, M123, M189, M190, M227, M362, M1, M131, M267, M306, M364, M262, MUT NS1, ASD7, Babawee, IR65482-7-216, IR 71033-121–15, MO1, MTU1010, IR26, IR40, IR66, Ndiang Marie, IR62, IR65482-136-2-2, 3K-19, 3K-47, 3K-53, 3K-132, 3K-168, 3K-187, 3K-200, 3K-290, and 3K-322
9.0	Highly susceptible	Telangana Sona, Utrirajappan, Balamwee, M272, M304, Milyang 63, ARC10550, Chinsaba, Pokkali, **BPT5204, and TN1 (checks)**

Bold genotypes are the checks used in the study.

### Identification of Candidate SNPs for BPH Resistance Using GWAS

**FIGURE 3:**
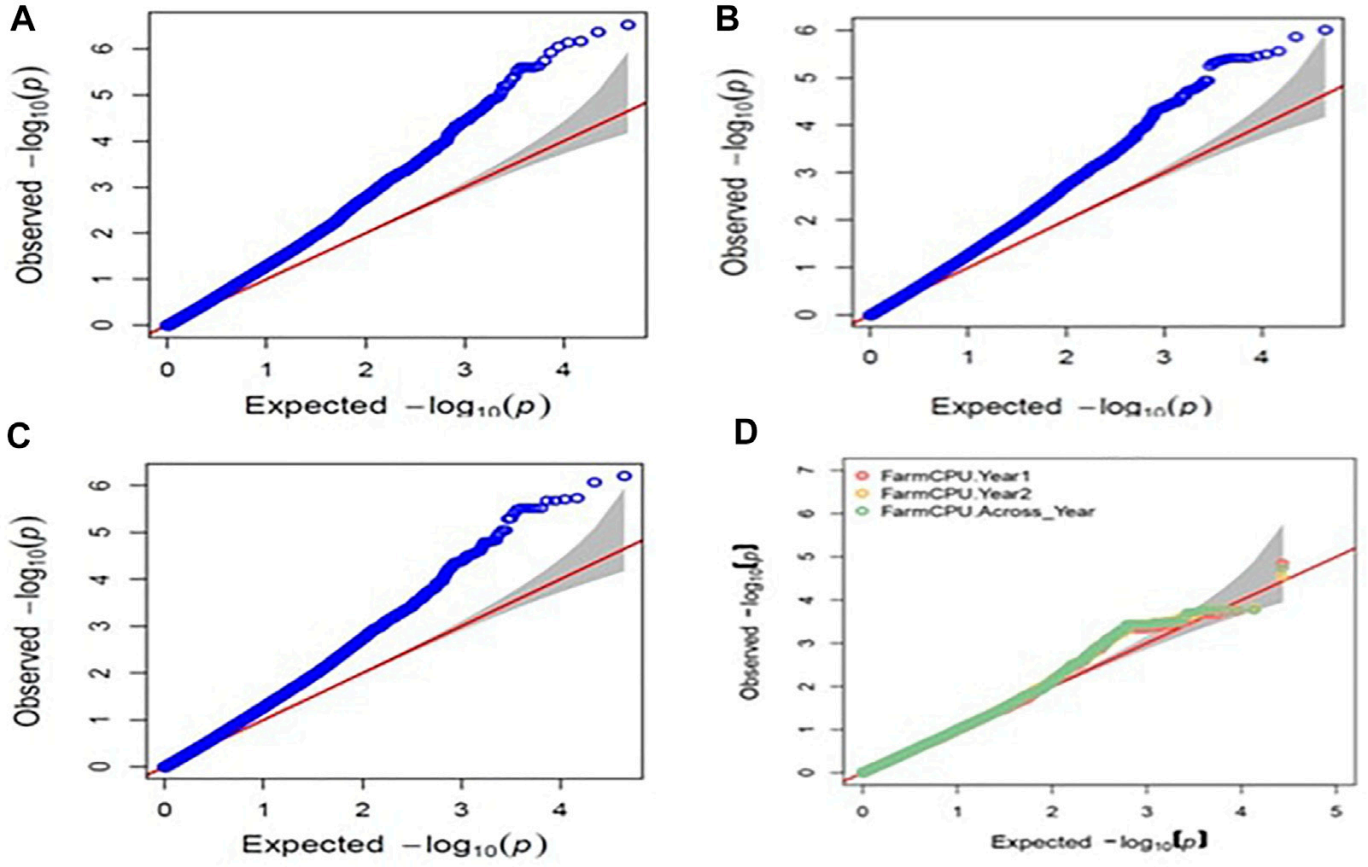
Q-Q plots obtained for BPH resistance in GWAS for **(A)** 2016 GLM model **(B)** 2017 GLM model **(C)** pooled years using GLM model **(D)** pooled years using FarmCPU model.

Raw genotyping data of the 391 MAGIC panel were used to perform the GWAS analysis which detected an aggregate of 27,000 polymorphic SNPs after curation of the data. The GWAS analysis using the BLUPs and cured polymorphic SNPs obtained from GBS data identified the common SNPs related to the resistance to BPH from three single-locus (GLM, MLM, and SUPER) and three multi-locus models (MLMM, BLINK, and FarmCPU). Manhattan plots (Supplementary Figures S2,S3) were used to display the *p*-value (0.001) distributions of SNPs with significant relationships across the chromosomes and the deviation of the observed statistical values from the expected statistical values was visualized in Q–Q plots (Figure 3). A total of 23 annotated stress-related SNPs linked to BPH resistance were identified from the GLM model on chromosomes 1, 2, and 6 from the model (Supplementary Table S4). Of the 23 annotated significant SNPs, 11 SNPs were found to cluster on chromosome 1 and were associated with six genes, viz., LOC_Os01g22640.1, LOC_Os01g23610.1, LOC_Os01g23680.1, LOC_Os01g23770.1, LOC_Os01g24050.1, and LOC_Os01g24950.1. These SNPs spanned from 12.7 to 14.0 Mb. One SNP (S2_5364800) was detected at a physical distance of 5.3 Mb on chromosome 2, while 11 significant SNPs identified on chromosome 6 were found to be associated with six genes viz.*,* LOC_Os06g07420.1, LOC_Os06g07620.1, LOC_Os06g15730.1, LOC_Os06g15740.1, LOC_Os06g15810.1, and LOC_Os06g15850.1. These SNPs were located between 3.5–3.6 Mb and 8.9–9.0 Mb.

While considering the results obtained from the GLM model (from the present study) and FarmCPU model from earlier studies (Satturu et al., 2020), it was observed that significant SNPs were identified at the same positions on chromosome 1 (1.3 Mb) and chromosome 6 (8.9 Mb). Of the total 54 significant SNPs identified from both the models, 20 SNPs (Table 6) were selected for genotyping in such a way to cover all the annotated genes representing functional defense-related mechanisms.

**TABLE 6:** List of 20 SNPs selected from GLM and FarmCPU models.

S. No.	SNP ID	SNP	Gene locus	Chr	Putative function
1	snpOS00912	S1_12737403	LOC_Os01g22640.1	1	GDSL-like lipase/acylhydrolase, putative, expressed
2	snpOS00913	S1_12742211	LOC_Os01g22660.1	1	GDSL-like lipase/acylhydrolase, putative, expressed
3	snpOS00914	S1_13365703	LOC_Os01g23770.1	1	OsMADS93 - MADS-box family gene with M-beta type-box, expressed
4	snpOS00915	S1_13898444	LOC_Os01g24690	1	60S ribosomal protein L23A, putative, expressed, response to abiotic stimulus, response to stress
5	snpOS00916	S2_5364800	LOC_Os02g10240.1	2	ZOS2-05 - C2H2 zinc finger protein, expressed
6	snpOS00917	S5_23249125	LOC_Os05g39590	5	AP2 domain-containing protein, expressed
7	snpOS00918	S5_23312204	LOC_Os05g39720	5	WRKY70, expressed
8	snpOS00919	S5_23314218	LOC_Os05g39720	5	WRKY70, expressed
9	snpOS00920	S6_5759360	LOC_Os06g11010	6	Eukaryotic aspartyl protease domain-containing protein, expressed
10	snpOS00921	S6_6514012	LOC_Os06g12160.1	6	AAA-type ATPase family protein, putative, expressed
11	snpOS00922	S6_6697070	LOC_Os06g12360.1	6	Pentatricopeptide, putative, expressed
12	snpOS00923	S6_7531433	LOC_Os06g13600.1	6	HEAT repeat family protein, putative, expressed
13	snpOS00924	S6_8914650	LOC_Os01g15830.1	6	Peroxidase precursor
14	snpOS00925	S6_8921200	LOC_Os01g15840.1	6	Pentatricopeptide repeat-containing protein
15	snpOS00926	S6_8932488	LOC_Os06g15750	6	NB-ARC domain-containing protein, expressed
16	snpOS00927	S6_8977190	LOC_Os06g15820.1	6	NHL repeat-containing protein, putative, expressed
17	snpOS00928	S6_8977949	LOC_Os06g15820.1	6	NHL repeat-containing protein, putative, expressed
18	snpOS00929	S6_8982135	LOC_Os06g15820.1	6	NHL repeat-containing protein, putative, expressed
19	snpOS00930	S6_9003866	LOC_Os06g15730	6	Nucleotide binding, response to stress, expressed
20	snpOS00931	S7_10995384	LOC_Os07g18600	7	OsFBL37-F-box domain and LRR-containing protein, expressed

### Annotated Candidate Genes Related to Resistance Against BPH

Among the four annotated genes on chromosome 1 (Table 6), LOC_Os01g24690 associated with SNP snpOS00915 was found to encode 60S ribosomal protein L23A, while LOC_Os01g22640.1 (snpOS00912) and LOC_Os01g22660.1 (snpOS00913) were associated with GDSL-like lipase/acylhydrolase protein and LOC_Os01g23770.1 (snpOS00914) with the OsMADS93-MADS-box family. LOC_Os02g10240.1 (snpOS00916) on chromosome 2 was found to encode the ZOS2-05-C2H2 zinc finger protein, while on chromosome 5, the significant SNPs, snpOS00917, snpOS00918, and snpOS00919 were found in the genic regions of LOC_Os05g39720 and LOC_Os05g39590 associated with the AP2 domain-containing protein and transcription factor-WRKY70. Furthermore, on chromosome 6, genes LOC_Os06g12360.1 and LOC_Os01g15840.1 co-located with snpOS00922 and snpOS00925, respectively, were found to be related to pentatricopeptide repeat proteins; SNPs snpOS00927, snpOS00928, and snpOS00929 (LOC_Os06g15820.1) with NHL repeat-containing protein, snpOS00926 was close to LOC_Os06g15750 with the NB-ARC functional ATPase protein, LOC_Os06g13600.1 (snpOS00923) was related to the HEAT repeat protein, while LOC_Os06g11010 (snpOS00920) and LOC_Os01g15830.1(snpOS00924) were linked to the eukaryotic aspartyl protease domain-containing protein and peroxidase precursors. On chromosome 7, LOC_Os07g18600 (snpOS00931) was found to be associated with the OsFBL37-F-box domain and LRR-containing protein.

KASP assays for these 20 SNPs were designed using 100 base pair sequences and were used in the validation studies along with five SNP markers specific to *Bph17*, *Bph32*, and *Bph9* available with the HTPG platform.

### Validation of Designed SNPs Associated With BPH Resistance

**FIGURE 4:**
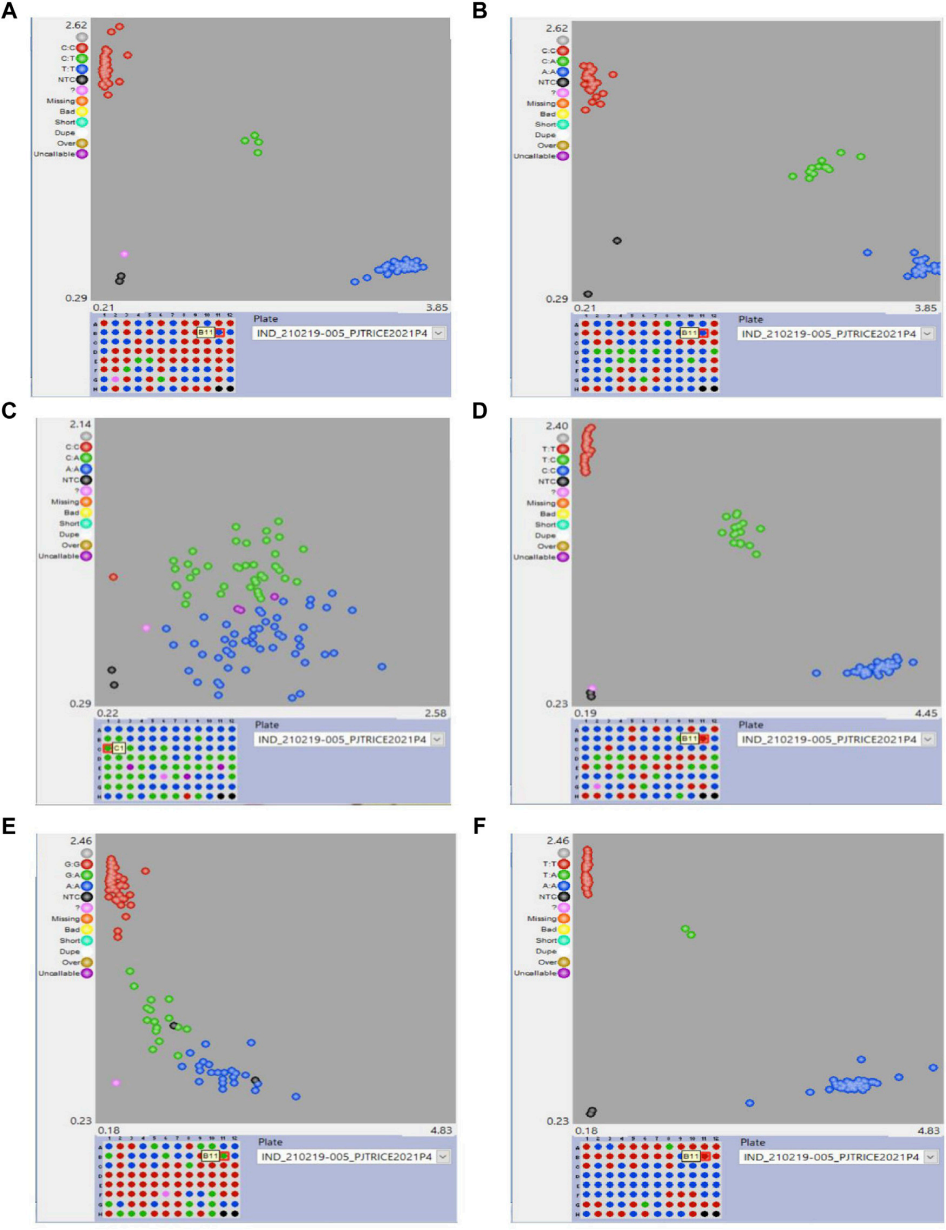
Clustering of genotypes for the SNPs **(A)** snpOS00922, **(B)** snpOS00923, **(C)** snpOS00912 **(D)** snpOS00915 **(E)** snpOS00929 and **(F)** snpOS00927.

Among the 20 designed SNPs for BPH resistance, six SNPs, viz, snpOS00912, snpOS00915, snpOS00922, snpOS00923, snpOS00927, and snpOS00929 were able to distinguish the resistant and susceptible checks in the presence and absence of favorable alleles, respectively (Table 7), while four SNPs (snpOS00913, snpOS00914, snpOS00916, and snpOS00925) were completely monomorphic (Supplementary Table S5). Among all the SNPs, snpOS00922 on chromosome 6 had been the most efficient marker with high power of distinguishing the genotypes into resistant and susceptible clusters. The resistant lines included M4, M88, M179, M192, M201, M229, M240, M284, M286, M344, IET23993, BM71, PTB33, 10-3, RathuHeenati, RathuHeenati accession, and RPV1355 with favorable allele T:T. snpOS00923, snpOS00912, and snpOS00929 pertaining to chromosome 6 differentiated resistant and susceptible genotypes in the presence and absence of favorable allele A:A, respectively (Figure 4). Correspondingly, snpOS00927and snpOS00915 had T:T and C:C favorable alleles, respectively, which also exhibited a moderate mode of demarcating the genotypes based on resistance reaction.

**TABLE 7:** Designed SNPs that were able to distinguish resistant and susceptible cultivars.

S. No.	Genotype	snpOS912	snpOS915	snpOS922	snpOS923	snpOS927	snpOS929
	Resistant lines						
1	PTB33	A:A	C:C	T:T	A:A	T:T	A:A
2	RathuHeenati	A:A	C:C	T:T	A:A	T:T	A:A
3	RP 2068-18-3-5	C:A	C:C	C:C	C:C	A:A	G:G
4	RP BIO 4918-230S	A:A	C:C	T:T	C:C	A:A	G:G
5	M4	A:A	T:C	T:T	C:C	A:A	G:G
6	M88	A:A	T:T	T:T	A:A	T:T	G:A
7	M179	A:A	C:C	T:T	A:A	T:T	G:A
8	M192	C:A	C:C	T:T	C:C	A:A	G:G
9	M201	A:A	T:T	T:T	A:A	T:T	G:A
10	M240	C:A	T:T	T:T	A:A	T:T	A:A
11	M284	C:A	C:C	T:T	A:A	T:T	A:A
12	M286	C:A	C:C	T:T	C:A	T:A	G:G
13	M312	C:A	C:C	C:C	C:C	A:A	G:G
14	M344	A:A	C:C	T:T	A:A	T:T	A:A
15	M359	C:A	C:C	C:C	C:C	A:A	G:G
16	IET23993	A:A	T:T	T:T	A:A	A:A	G:G
17	BM71	A:A	T:T	T:T	C:C	T:T	G:A
18	RPV1355	?	T:T	T:T	A:A	A:A	G:G
19	M229	A:A	C:C	T:T	C:C	A:A	G:G
20	10-3	A:A	C:C	T:T	C:C	A:A	G:G
	Susceptible lines						
1	TN1	C:C	C:C	C:C	C:C	A:A	G:G
2	BPT5204	C:C	C:C	C:C	C:C	A:A	G:G
3	KNM118	C:A	C:C	T:T	A:A	T:T	?
4	Telangana Sona	C:A	T:T	C:C	A:A	A:A	G:G
5	ASD 7	A:A	C:C	T:T	A:A	A:A	G:G
6	Utrirajappan	A:A	C:C	T:T	C:C	A:A	G:G
7	Balamwee	A:A	C:C	T:T	A:A	T:T	G:A
8	M1	A:A	C:C	T:T	A:A	T:T	G:A
9	M123	A:A	C:C	T:T	C:C	T:T	A:A
10	M131	A:A	C:C	T:T	A:A	T:T	G:A
11	M189	C:C	C:C	C:C	C:A	T:T	G:A
12	M190	C:C	?	C:C	C:A	?	A:A
13	M227	A:A	T:T	C:C	C:C	A:A	G:G
14	M267	A:A	T:T	T:T	A:A	T:T	G:A
15	M272	A:A	C:C	T:T	A:A	T:T	A:A
16	M304	A:A	T:C	C:T	C:A	T:A	G:G
17	M306	A:A	C:C	T:T	A:A	T:T	G:A
18	M362	C:A	C:C	T:T	A:A	T:T	A:A
19	M364	A:A	T:T	C:C	A:A	T:T	A:A

### Validation of the SNPs Specific to *Bph17*, *Bph32*, and *Bph9* in a Pre-Defined Panel of Genotypes

**FIGURE 5:**
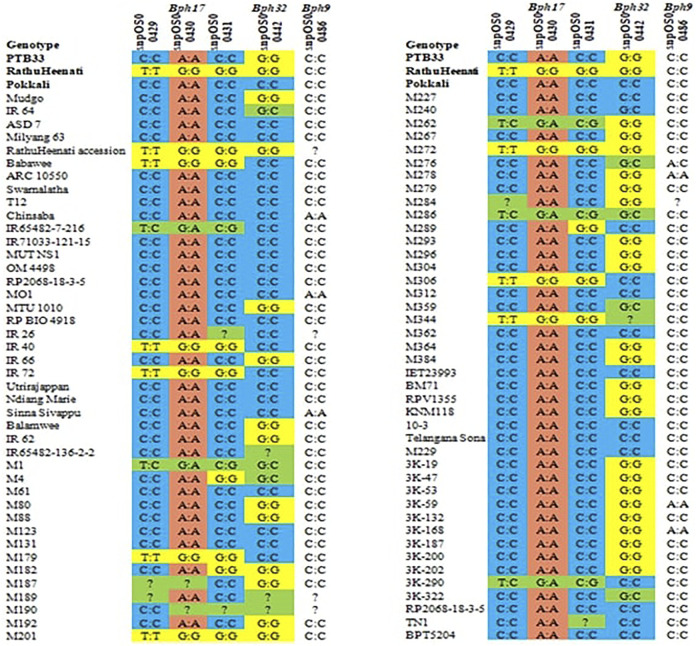
Validation of *Bph17*, *Bph32* and *Bph9* SNPs in genotypes.

A total of five SNP markers reported to be specific to *Bph17*, *Bph32*, and *Bph9* genes were used for validating the panel of genotypes for BPH resistance (Figure 5). Of the 83 genotypes and checks evaluated for the *Bph17* gene, 10 lines were having the favorable alleles for snpOS00429 (T:T) and snpOS00430 (G:G), while the favorable allele (G:G) for snpOS00431 was present in nine genotypes (Supplementary Table S6). Altogether, five MAGIC lines (M201, M179, M306, M272, and M344) and four gene differentials (RathuHeenati, RathuHeenati accession, Babawee, and IR72) were positive for all the three *Bph17* gene-specific SNPs. Lines M1, M262, M286, and 3K-290 and gene differential IR65482-7-216 were heterozygous for these loci. Regarding the *Bph32* gene, 38 accessions including 17 MAGIC lines, seven gene differentials, and nine 3K lines along with PTB33, KNM118, RDR1200, RPV1355, and BM71 were detected to contain the positive allele (G:G) in homozygous condition (Supplementary Table S7). The original donor PTB33 was found with the positive allele for the *Bph32* gene while RathuHeenati and RathuHeenati accession confirmed the presence of *Bph17* and *Bph32* genes.

Three MAGIC lines viz., M201, M272, and M344 had the favorable alleles for both *Bph17* and *Bph32* genes (Table 8). MAGIC lines M1 and M286 were noted to be heterozygous for both the gene loci (*Bph17* and *Bph32*)*.* Although the SNP for *Bph9* (snpOS00486) was detected in lines M278, 3K-18, 3K-41, 3K-59, 3K-60, 3K-80, 3K-168, 3K-182, 3K-184, 3K-292, 3K-321, SinnaSivappu, MO1, and Chinsaba (Supplementary Table S8) with favorable allele A:A, its absence in the original donor, Pokkali, determines its ineffectiveness in working as a diagnostic marker as observed in the present study.

**TABLE 8:** List of functional SNPs and favorable alleles specific to *Bph17* and *Bph32* genes validated in the panel of genotypes.

S. No.	Genotypes possessing favorable allele	SNPs and their favorable alleles specific to BPH genes			
		*Bph17-*snpOS00429 (T:T)	*Bph17-*snpOS00430 (G:G)	*Bph17-*snpOS00431 (G:G)	*Bph32-*snpOS00442 (G:G)
	MAGIC lines				
1	M1	T:C	G:A	C:G	G:C
2	M179	T:T	G:G	G:G	C:C
3	M201	T:T	G:G	G:G	G:G
4	M262	T:C	G:A	C:G	G:G
5	M272	T:T	G:G	G:G	G:G
6	M286	T:C	G:A	C:G	G:C
7	M306	T:T	G:G	G:G	C:C
8	M344	T:T	G:G	G:G	G:G
	Gene differentials				
9	RathuHeenati	T:T	G:G	G:G	G:G
10	Balamwee	T:T	G:G	G:G	C:C
11	RathuHeenati accession	T:T	G:G	G:G	G:G
12	IR72	T:T	G:G	G:G	C:C
13	IR40	T:T	G:G	C:G	C:C
14	IR 65482-7-216	T:C	G:A	NA	C:C

## Discussion

The brown planthopper is one of the utmost devastating rice pests, producing significant crop losses. Identification of resistant donors and effective screening approaches for evaluating breeding lines are essential for transferring BPH resistance genes into high-yielding cultivars. In addition, a high amount of genetic diversity lowers the risk of widespread insect epidemics (Newton et al., 2009). The most often used technique at the IRRI and by the National Agricultural Research and Extension Systems (NARES) has been the mass screening of the BPH involving the evaluation of rice genotypes at the seedling stage. Using SSST, the IRRI evaluated many germplasm lines for resistance to three BPH biotypes as mentioned by Jackson (1997). SSST is a rapid and most widely used mass screening method for evaluating seedling susceptibility to the BPH (Fujita et al., 2013; Horgan et al., 2015). In the present study, ANOVA disclosed a significant mean sum of squares for BPH damage scores for different sources of variation. The block effect (unadjusted) and the treatment effects (adjusted and unadjusted) were significant for indicating the presence of a considerable amount of genetic variability in the material studied. Similarly, the effects due to checks, varieties, and checks versus varieties were significant, suggesting the test entries to be significantly different from the checks. The adjusted block effects were non-significant for BPH damage scores indicating homogeneity of evaluation blocks.

The phenotypic responses of the 83 genotypes with checks indicated varying levels of resistance reaction to BPH screening. A comparison of the damage scores of both seasons revealed 13 genotypes as resistant to the BPH with a low damage score extending from 1.3 to 3.0. The resistant lines that comprised gene differentials, RathuHeenati, RP 2068-18-3-5, and RP Bio4918-230S were previously reported to be resistant (Sunil et al., 2018; Akanksha et al., 2019) along with nine MAGIC lines (Durga Sai, 2017). Susceptible checks TN1 and BPT5204 exhibited absolute susceptibility (9.0 score), while the resistant checks PTB33, RathuHeenati, and RP 2068-18-3-5 revealed damage scores of ≤2.0. Several researchers reported PTB33 as resistant to BPH (Harini et al., 2013; Ramulamma et al*.,* 2015; Reddy et al., 2016; Thamarai and Soundararajan, 2017; Udayasree et al.*,* 2018), along with RathuHeenati and RP 2068-18-3-5 that were reported to exhibit durable resistance by Sarao and Bentur (2016); Sunil et al. (2018) and Akanksha et al*.* (2019). Similar studies were also taken up by Gangaram et al*.* (2019) who evaluated 74 rice genotypes of Sikkim and Tripura against the BPH in glasshouse conditions along with resistant (PTB33) and susceptible (TN1) checks. The polarity in the resistance response of the rice accessions might be due to the variation in the toxin or antibiotic produced by the rice plant (Qiu et al., 2011; Singh et al., 2019), notably alkaloids or other organic compounds that have repellent effects against the BPH (Sodiq, 2009; Qiu et al., 2011), thus exhibiting varying levels of reaction to the BPH infestation. Furthermore, a thorough investigation of their HPR mechanisms is indispensable to uncover knowledge about the types of resistance, such as antibiosis, antixenosis, and tolerance in each of the genotypes. The production of resistant cultivars that can withstand and prevail over BPH damage in the field for longer periods of time with fewer pesticide applications will result from the research-based application of these HPR mechanisms.

The GWAS is an efficient and reliable method for the excavation of genetic loci and candidate genes that are accountable for natural variability in a polygenic trait (Ishimaru et al., 2013). The density of molecular markers, population size, trait of interest, phenotype-based evaluation, and statistical techniques are the factors that influence the power of GWAS to find related loci for a target trait (Zhang et al., 2015). In the present investigation, based on the consistency and power of associations, 23 corresponding significant SNPs found by the GLM statistical approach were considered for further annotations. The 11 SNPs associated with six candidate genes on chromosome 1 were just 6 Mb away from the reported *Bph38(t)* that is located between 20.7 and 21.2 Mb (Balachiranjeevi et al*.,* 2019), while a single SNP on chromosome 2 was detected at a physical distance of 5.3 Mb away from the reported *Bph13(t)* gene located at 31 Mb (Liu et al*.*, 2001), *Qbph2* at 22 Mb (Sun et al*.,* 2007), and *qBph2* at 17 Mb (Huang et al*.,* 2012). Similarly, 11 significant SNPs identified on chromosome 6 were in close proximity to *Bph22(t)* located at 3.4 Mb (Harini et al*.*, 2010), *Bph3* and *bph4* (1.4–1.6 Mb; Jairin et al., 2007, 2010), *Bph20(t)* (9.3 Mb; Rahman et al*.*, 2009)*, bph29* (5.3Mb; Wang et al*.*, 2015), *bph25(t)* (1.7 Mb; Myint et al*.,* 2012), and *Bph32* (1.2–1.5 Mb; Ren et al*.*, 2016). Co-localization of the SNPs that were identified to be significant in close proximity to the reported resistance genes/QTLs such as *Bph22(t)* and *Bph20(t)* provides a clue about the relatedness of the parents of these MAGIC lines with the original BPH gene differentials carrying these genes/QTLs.

Primarily, of the 42 BPH genes reported, 14 genes have been cloned and characterized to expedite the development of broad-spectrum and durable insect-resistant rice varieties (Du et al., 2020). These genes code for proteins that detect insect effectors and activate defense-related pathways. Similarly, several genes identified in the present investigation have functions pertinent to BPH resistance. The 60S ribosomal protein L23A encoded by LOC_Os01g24690 (snpOS00915) was found to have multiple functions and differential regulation during stress conditions as suggested by Fromont-Racine et al. (2003). GDSL-like lipase/acylhydrolase associated with LOC_Os01g22640.1 (snpOS00912) and LOC_Os01g22660.1 (snpOS00913) acts as an elicitor of systemic resistance through ethylene signaling, thus playing a critical part for both local and systemic resistance in plant immunity systems (Kwon et al., 2010). These family genes were found to be significantly increased in pest-infested leaves and were correlated with different kinds of biotic stress responses (Truong et al., 2017). Similarly, LOC_Os01g23770.1 (snpOS00914) encoding the OsMADS93-MADS-box family gene protein was related to abiotic stress tolerance (Castelán-Munoz et al., 2019), while LOC_Os02g10240.1 (snpOS00916) on chromosome 2 was found to encode the ZOS2-05-C2H2 zinc finger protein that functions as a transcriptional activator in regulating abiotic stress signaling pathways as mentioned by Han et al. (2020). LOC_Os05g39590 and LOC_Os05g39720 (snpOS00917, snpOS00918, and snpOS00919) on chromosome 5 associated with AP2 domain-containing protein and WRKY70 transcription factor were found to be engaged in a stress-tolerant system with the control over ABA-dependent/independent stress-responsive pathways (Eulgem and Somssich, 2007; Khong et al., 2008) that could play a major role in BPH resistance. A similar class of transcription factors (WRKY46 and WRKY72) responsible for BPH resistance were reported by Hu et al*.* (2017) for the *Bph14* gene, which, by over-expressing RLCK281 and callose synthase genes coupled with trypsin secretion, caused the inhibition of phloem sucking, thereby providing resistance to the BPH.

In the same way, LOC_Os06g12360.1 (snpOS00922) and LOC_Os01g15840.1 (snpOS00925) were associated with pentatricopeptide repeat proteins that have implications as mitochondrion-localized proteins in the defense against biotic pathogens (Laluk et al., 2011). LOC_Os06g15820.1 (snpOS00927, snpOS00928, and snpOS00929) associated with the NHL repeat-containing protein was found to encode a plasma membrane protein whose over-expression was correlated with increased resistance to biotic stress as reported by Varet et al. (2003), and LOC_Os06g15750 (snpOS00926) with the NB-ARC functional ATPase protein was found possessing regulatory activity of the R protein that triggers the induction of plant defenses to restrict pathogen proliferation achieving resistance as stated by Ooijen et al. (2008). LOC_Os06g13600.1 gene (snpOS00923) related to the HEAT repeat protein has been identified to play a critical part in the immunity of plant systems by facilitating protein–protein interactions (Monaghan and Li, 2010), while LOC_Os06g11010 (snpOS00920) and LOC_Os01g15830.1(snpOS00924) were associated with the eukaryotic aspartyl protease domain-containing protein and peroxidase precursors that might be useful in providing resistance to the BPH. LOC_Os07g18600 (snpOS00931) on chromosome 7 was linked to the OsFBL37-F-box domain and LRR-containing protein that aids in recognizing pathogen-associated molecular patterns or effectors and turning on the host-resistance pathways. A similar protein conferring resistance to the BPH was reported for Bph26, *Bph18*, and *Bph9* genes that acted as a sensor for signal transduction in response to BPH attack and inhibiting the pest from sucking the phloem sap (Tamura et al., 2014; Ji et al., 2016). Annotation works such as these may benefit researchers by intuiting the genic level of rice-BPH interactions along with the production of new insect-resistant rice cultivars, resulting in better long-term control of the BPH.

To validate the suitability of the set of SNPs, these were converted into a KASP assay, which is a uniplex SNP genotyping platform. Previous research has been reported on the generation of KASP markers in a variety of crop species, including rice, wheat, maize, bajra, sorghum, tomato, potato, cotton, peanut, and rubus species (Rasheed et al., 2016; Zhao et al., 2017; Ryu et al., 2018; Steele et al., 2018; Burow et al., 2019; Devran and Kahveci, 2019; Chen et al., 2021; Kante et al., 2021). A total of six of our designed SNPs were able to distinguish the resistant and susceptible lines, which correlated with our previous phenotyping results (Lakshmi et al., 2021). The SNPs were associated with defense-related genes with putative functions viz.*,* GDSL-like lipase/acylhydrolase, 60S ribosomal protein, pentatricopeptide protein, HEAT repeat family protein, and NHL repeat-containing protein. Altogether, these designed SNPs having functional defense mechanisms and moderate efficiency in classifying the BPH-resistant and susceptible genotypes could be used as diagnostic markers in MAB studies after validating them in biparental populations developed for BPH resistance.

The BPH biotype 4 is widely spread across Asian countries for which *Bph17* and *Bph32* genes were found to be effective (Liu et al., 2015; Ren et al., 2016). *Bph17* was found to possess three tandem duplicated genes encoding for plasma membrane-localized lectin receptor kinase. Analysis of the sequence revealed the polymorphism in a single nucleotide of three genes (OsLecRK1, OsLecRK2, and OsLecRK3) to be accountable for controlling resistance in RathuHeenati (Liu et al., 2015). Similarly, *Bph32* encodes an unknown protein holding a signal peptide and SCOP d1gkna2 domain (Ren et al., 2016). SNPs associated with these genes contained positive alleles in the original donors PTB33 (*Bph32*) and RathuHeenati (*Bph17* and *Bph32*), confirming the presence of these loci as reported in previous studies by Liu et al. (2015), Ren et al. (2016), Jena et al. (2017), and Kusumawati et al. (2018). As the combination of multiple BPH-specific genes is an effective strategy to develop cultivars with broad-spectrum resistance, the five digenic lines viz, M201, M272, M344, RathuHeenati, and RathuHeenati accession with *Bph17* and *Bph32* genes could also be used for introducing BPH resistance into popular varieties through the MAB approach. Also, these lines are highly useful for studying the effects of each gene either singly or in combination with yield and other agronomic traits.

## Conclusion

The present study reported the identification of BPH-resistant donors by phenotypic screening followed by validation with BPH-related SNPs that could contribute to the development of new BPH-resistant cultivars. Screening of germplasm lines for BPH resistance identified 13 genotypes as the best with a low damage score comparable to the resistant checks. Molecular and morphological validation of the germplasm lines and checks with the reported SNPs pinned down MAGIC lines, M201, M272, and M344 for having favorable alleles for both *Bph17* and *Bph32,* indicating their use as donors for introducing BPH resistance. Since the gene-specific markers for *Bph17* and *Bph32* which proved to be effective against Asian biotype 4 were validated in the original donors PTB33 and RathuHeenati, the purity of the lines maintained was confirmed. Accompanying these reported SNPs, validation with our designed SNPs has successfully identified six robust SNP markers that can distinguish the genotypes based on resistance reaction to the BPH and could be used as diagnostic markers for their routine use in rice improvement programs targeting BPH resistance.

### Remarks

With respect to the genotypes used in the study, RathuHeenati is a Sri Lankan *indica* rice cultivar, while RathuHeenati accession is a derived line of the original RathuHeenati cultivar.

## Data Availability

The original contributions presented in the study are included in the article/Supplementary Material, further inquiries can be directed to the corresponding author.
