# Supplementary material for: Development and Validation of Diagnostic KASP Markers for Brown Planthopper Resistance in Rice
Source: Front Genet. 2022 Jul 8;13:914131. doi: 10.3389/fgene.2022.914131 (PMC9309266; doi:10.3389/fgene.2022.914131)
Supplement: Supplementary file 1 [file DataSheet2.docx]

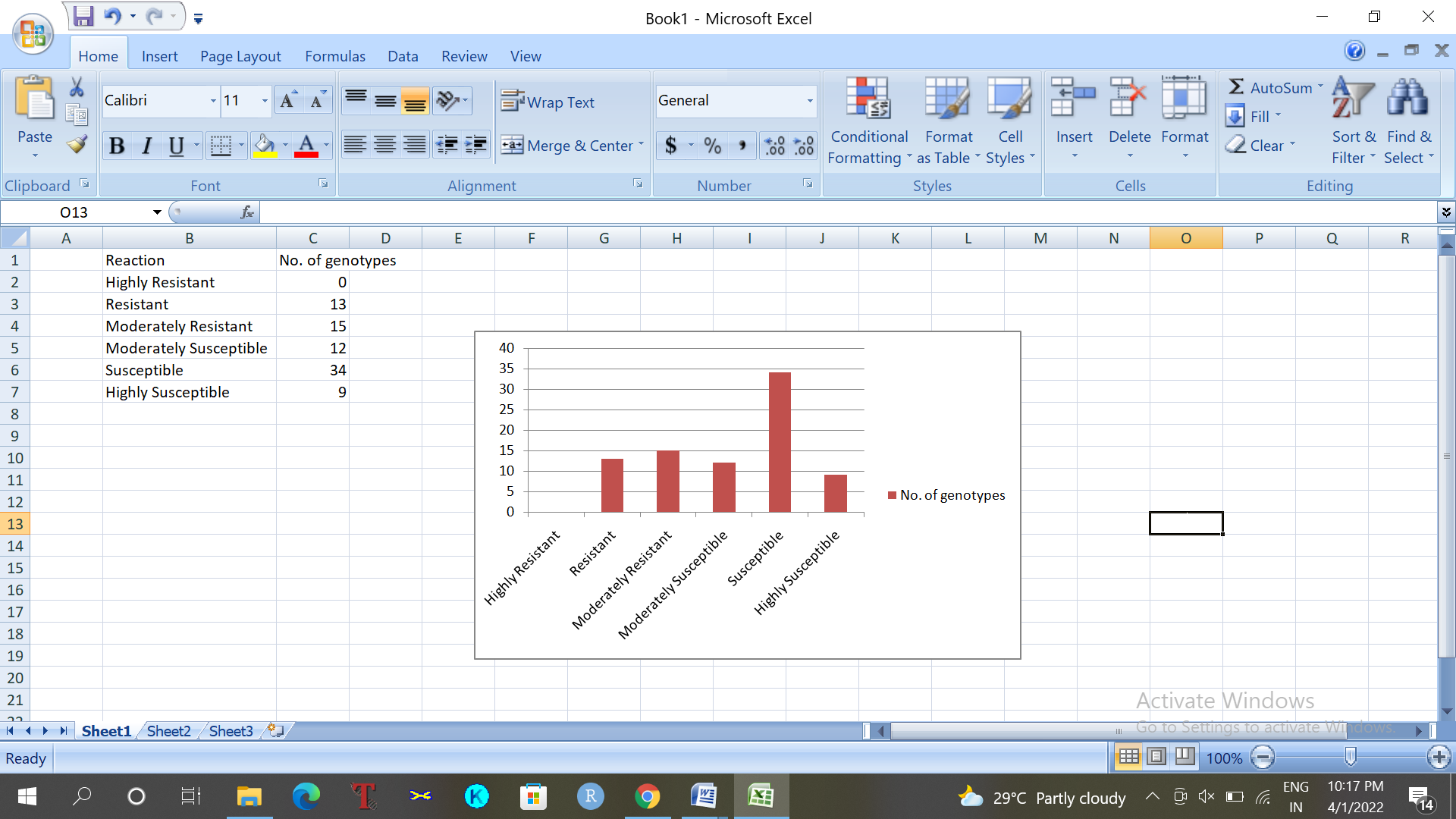


**Supplementary Fig. S1. Histogram depicting the number of genotypes in each class of BPH reaction for both the seasons**

**
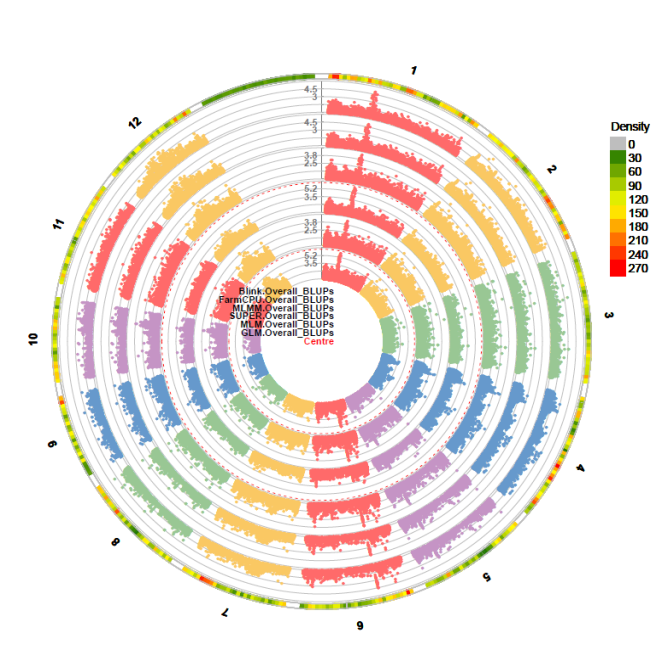
**

**Supplementary Fig. S2. Circular-Manhattan plot of (a) GLM (b) MLM (c) SUPER (d) MLMM (e) FarmCPU and (f) Blink model (from the centre) showing the significant SNP associations (*p* <0.001) on the chromosomes with BPH resistance for the pooled data**

**
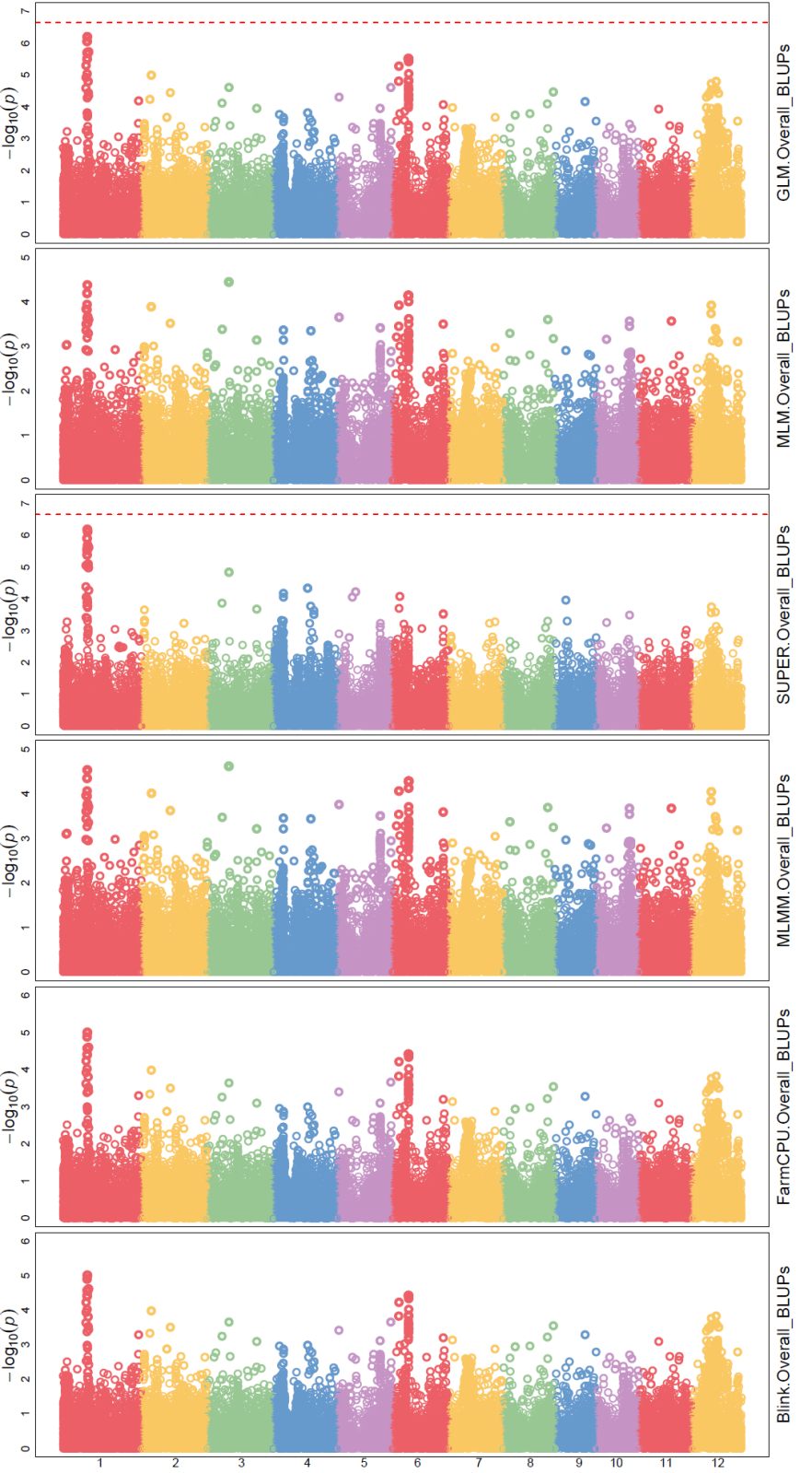
**

**(a)**

**
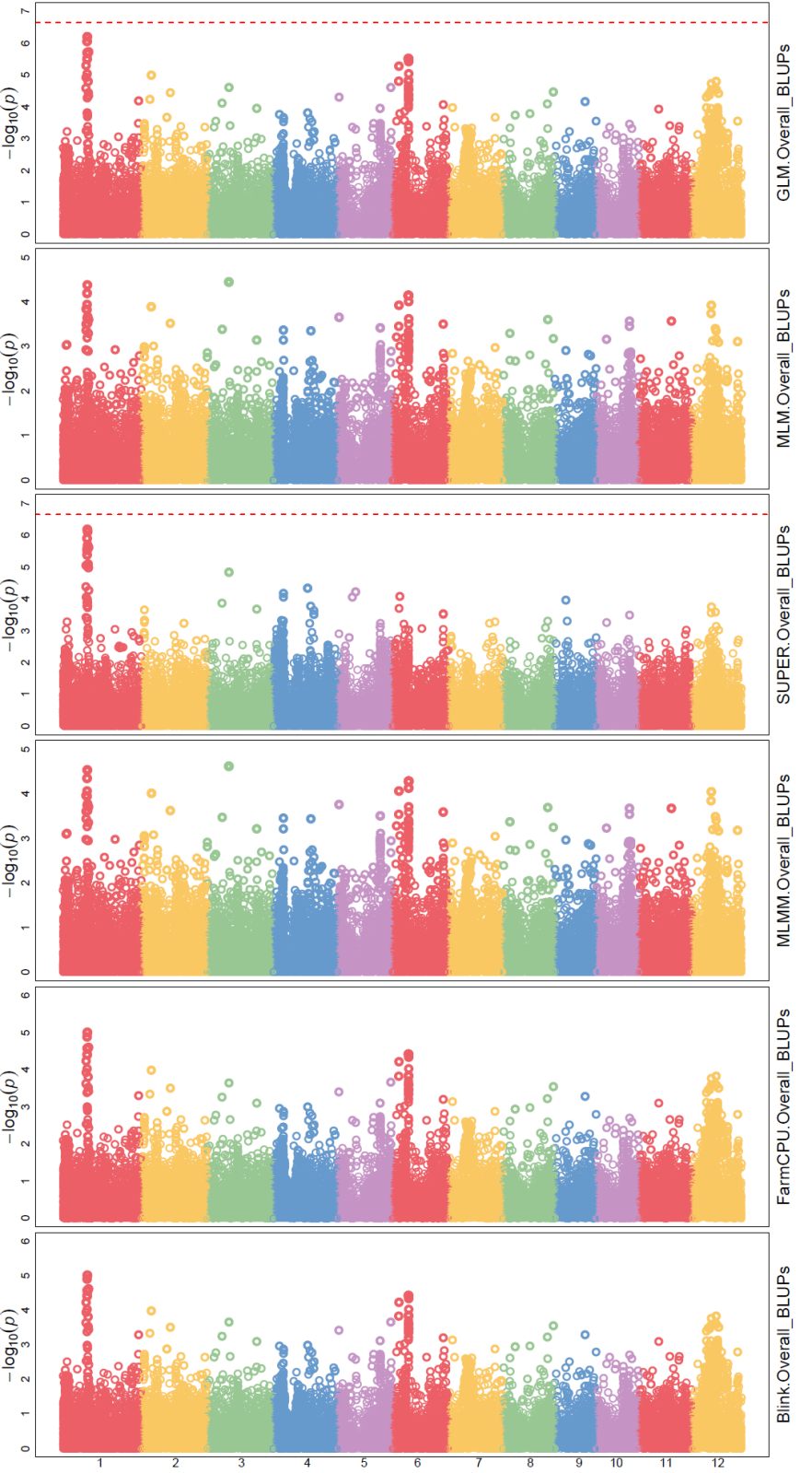
**

**(b)**

**Supplementary Fig. S3. Manhattan plots of the selected consistent models (a) GLM and (b) FarmCPU with significant SNPs detected for BPH resistance in both the years**
