# Supplementary material for: Development and Validation of Diagnostic KASP Markers for Brown Planthopper Resistance in Rice
Source: Front Genet. 2022 Jul 8;13:914131. doi: 10.3389/fgene.2022.914131 (PMC9309266; doi:10.3389/fgene.2022.914131)
Supplement: Supplementary file 2 [file DataSheet1.docx]

| **S.No.** | **Lines** | **Code** | **S.No.** | **Lines** | **Code** | **S.No.** | **Lines** | **Code** | **S.No.** | **Lines** | **Code** |
| --- | --- | --- | --- | --- | --- | --- | --- | --- | --- | --- | --- |
| 1 | MIB-4728 | **M1** | 46 | MIB-4832 | **M46** | 91 | MIB-5036 | **M91** | 136 | MIB-5238 | **M136** |
| 2 | MIB-4734 | **M2** | 47 | MIB-4833 | **M47** | 92 | MIB-5054 | **M92** | 137 | MIB-5240 | **M137** |
| 3 | MIB-4736 | **M3** | 48 | MIB-4834 | **M48** | 93 | MIB-5056 | **M93** | 138 | MIB-5245 | **M138** |
| 4 | MIB-4737 | **M4** | 49 | MIB-4835 | **M49** | 94 | MIB-5060 | **M94** | 139 | MIB-5246 | **M139** |
| 5 | MIB-4742 | **M5** | 50 | MIB-4836 | **M50** | 95 | MIB-5065 | **M95** | 140 | MIB-5248 | **M140** |
| 6 | MIB-4743 | **M6** | 51 | MIB-4853 | **M51** | 96 | MIB-5067 | **M96** | 141 | MIB-5250 | **M141** |
| 7 | MIB-4744 | **M7** | 52 | MIB-4860 | **M52** | 97 | MIB-5070 | **M97** | 142 | MIB-5252 | **M142** |
| 8 | MIB-4745 | **M8** | 53 | MIB-4863 | **M53** | 98 | MIB-5071 | **M98** | 143 | MIB-5255 | **M143** |
| 9 | MIB-4747 | **M9** | 54 | MIB-4866 | **M54** | 99 | MIB-5072 | **M99** | 144 | MIB-5257 | **M144** |
| 10 | MIB-4749 | **M10** | 55 | MIB-4867 | **M55** | 100 | MIB-5097 | **M100** | 145 | MIB-5262 | **M145** |
| 11 | MIB-4750 | **M11** | 56 | MIB-4868 | **M56** | 101 | MIB-5099 | **M101** | 146 | MIB-5266 | **M146** |
| 12 | MIB-4751 | **M12** | 57 | MIB-4874 | **M57** | 102 | MIB-5102 | **M102** | 147 | MIB-5268 | **M147** |
| 13 | MIB-4755 | **M13** | 58 | MIB-4876 | **M58** | 103 | MIB-5104 | **M103** | 148 | MIB-5269 | **M148** |
| 14 | MIB-4760 | **M14** | 59 | MIB-4877 | **M59** | 104 | MIB-5113 | **M104** | 149 | MIB-5271 | **M149** |
| 15 | MIB-4762 | **M15** | 60 | MIB-4881 | **M60** | 105 | MIB-5117 | **M105** | 150 | MIB-5272 | **M150** |
| 16 | MIB-4766 | **M16** | 61 | MIB-4882 | **M61** | 106 | MIB-5123 | **M106** | 151 | MIB-5274 | **M151** |
| 17 | MIB-4767 | **M17** | 62 | MIB-4884 | **M62** | 107 | MIB-5128 | **M107** | 152 | MIB-5281 | **M152** |
| 18 | MIB-4769 | **M18** | 63 | MIB-4900 | **M63** | 108 | MIB-5129 | **M108** | 153 | MIB-5286 | **M153** |
| 19 | MIB-4770 | **M19** | 64 | MIB-4901 | **M64** | 109 | MIB-5138 | **M109** | 154 | MIB-5287 | **M154** |
| 20 | MIB-4774 | **M20** | 65 | MIB-4922 | **M65** | 110 | MIB-5139 | **M110** | 155 | MIB-5289 | **M155** |
| 21 | MIB-4776 | **M21** | 66 | MIB-4923 | **M66** | 111 | MIB-5143 | **M111** | 156 | MIB-5290 | **M156** |
| 22 | MIB-4778 | **M22** | 67 | MIB-4926 | **M67** | 112 | MIB-5147 | **M112** | 157 | MIB-5292 | **M157** |
| 23 | MIB-4781 | **M23** | 68 | MIB-4931 | **M68** | 113 | MIB-5149 | **M113** | 158 | MIB-5293 | **M158** |
| 24 | MIB-4784 | **M24** | 69 | MIB-4932 | **M69** | 114 | MIB-5151 | **M114** | 159 | MIB-5295 | **M159** |
| 25 | MIB-4785 | **M25** | 70 | MIB-4934 | **M70** | 115 | MIB-5154 | **M115** | 160 | MIB-5306 | **M160** |
| 26 | MIB-4794 | **M26** | 71 | MIB-4948 | **M71** | 116 | MIB-5156 | **M116** | 161 | MIB-5310 | **M161** |
| 27 | MIB-4795 | **M27** | 72 | MIB-4949 | **M72** | 117 | MIB-5158 | **M117** | 162 | MIB-5312 | **M162** |
| 28 | MIB-4797 | **M28** | 73 | MIB-4954 | **M73** | 118 | MIB-5159 | **M118** | 163 | MIB-5323 | **M163** |
| 29 | MIB-4799 | **M29** | 74 | MIB-4957 | **M74** | 119 | MIB-5164 | **M119** | 164 | MIB-5325 | **M164** |
| 30 | MIB-4802 | **M30** | 75 | MIB-4958 | **M75** | 120 | MIB-5166 | **M120** | 165 | MIB-5328 | **M165** |
| 31 | MIB-4803 | **M31** | 76 | MIB-4964 | **M76** | 121 | MIB-5168 | **M121** | 166 | MIB-5341 | **M166** |
| 32 | MIB-4804 | **M32** | 77 | MIB-4965 | **M77** | 122 | MIB-5174 | **M122** | 167 | MIB-5364 | **M167** |
| 33 | MIB-4805 | **M33** | 78 | MIB-4972 | **M78** | 123 | MIB-5178 | **M123** | 168 | MIB-5370 | **M168** |
| 34 | MIB-4806 | **M34** | 79 | MIB-4973 | **M79** | 124 | MIB-5180 | **M124** | 169 | MIB-5373 | **M169** |
| 35 | MIB-4807 | **M35** | 80 | MIB-4996 | **M80** | 125 | MIB-5181 | **M125** | 170 | MIB-5374 | **M170** |
| 36 | MIB-4808 | **M36** | 81 | MIB-4997 | **M81** | 126 | MIB-5193 | **M126** | 171 | MIB-5375 | **M171** |
| 37 | MIB-4809 | **M37** | 82 | MIB-5000 | **M82** | 127 | MIB-5196 | **M127** | 172 | MIB-5377 | **M172** |
| 38 | MIB-4811 | **M38** | 83 | MIB-5003 | **M83** | 128 | MIB-5200 | **M128** | 173 | MIB-5378 | **M173** |
| 39 | MIB-4812 | **M39** | 84 | MIB-5005 | **M84** | 129 | MIB-5201 | **M129** | 174 | MIB-5385 | **M174** |
| 40 | MIB-4815 | **M40** | 85 | MIB-5014 | **M85** | 130 | MIB-5205 | **M130** | 175 | MIB-5387 | **M175** |
| 41 | MIB-4816 | **M41** | 86 | MIB-5016 | **M86** | 131 | MIB-5208 | **M131** | 176 | MIB-5393 | **M176** |
| 42 | MIB-4822 | **M42** | 87 | MIB-5019 | **M87** | 132 | MIB-5225 | **M132** | 177 | MIB-5394 | **M177** |
| 43 | MIB-4823 | **M43** | 88 | MIB-5021 | **M88** | 133 | MIB-5226 | **M133** | 178 | MIB-5395 | **M178** |
| 44 | MIB-4824 | **M44** | 89 | MIB-5023 | **M89** | 134 | MIB-5229 | **M134** | 179 | MIB-5397 | **M179** |
| 45 | MIB-4831 | **M45** | 90 | MIB-5035 | **M90** | 135 | MIB-5236 | **M135** | 180 | MIB-5398 | **M180** |

**Supplementary Table S1. List of 391 MAGIC *indica* lines used for Genome Wide SNP**

**discovery**

| **S.No.** | **Lines** | **Code** | **S.No.** | **Lines** | **Code** | **S.No.** | **Lines** | **Code** | **S.No.** | **Lines** | **Code** |
| --- | --- | --- | --- | --- | --- | --- | --- | --- | --- | --- | --- |
| 181 | MIB-5400 | **M181** | 226 | MIB-5557 | **M226** | 271 | MIB-5685 | **M271** | 316 | MIB-5814 | **M316** |
| 182 | MIB-5405 | **M182** | 227 | MIB-5558 | **M227** | 272 | MIB-5687 | **M272** | 317 | MIB-5818 | **M317** |
| 183 | MIB-5406 | **M183** | 228 | MIB-5563 | **M228** | 273 | MIB-5689 | **M273** | 318 | MIB-5823 | **M318** |
| 184 | MIB-5409 | **M184** | 229 | MIB-5573 | **M229** | 274 | MIB-5691 | **M274** | 319 | MIB-5827 | **M319** |
| 185 | MIB-5418 | **M185** | 230 | MIB-5574 | **M230** | 275 | MIB-5692 | **M275** | 320 | MIB-5828 | **M320** |
| 186 | MIB-5449 | **M186** | 231 | MIB-5583 | **M231** | 276 | MIB-5694 | **M276** | 321 | MIB-5829 | **M321** |
| 187 | MIB-5450 | **M187** | 232 | MIB-5587 | **M232** | 277 | MIB-5696 | **M277** | 322 | MIB-5835 | **M322** |
| 188 | MIB-5454 | **M188** | 233 | MIB-5589 | **M233** | 278 | MIB-5698 | **M278** | 323 | MIB-5837 | **M323** |
| 189 | MIB-5457 | **M189** | 234 | MIB-5592 | **M234** | 279 | MIB-5701 | **M279** | 324 | MIB-5839 | **M324** |
| 190 | MIB-5458 | **M190** | 235 | MIB-5596 | **M235** | 280 | MIB-5705 | **M280** | 325 | MIB-5841 | **M325** |
| 191 | MIB-5459 | **M191** | 236 | MIB-5601 | **M236** | 281 | MIB-5709 | **M281** | 326 | MIB-5845 | **M326** |
| 192 | MIB-5460 | **M192** | 237 | MIB-5602 | **M237** | 282 | MIB-5710 | **M282** | 327 | MIB-5850 | **M327** |
| 193 | MIB-5465 | **M193** | 238 | MIB-5608 | **M238** | 283 | MIB-5714 | **M283** | 328 | MIB-5853 | **M328** |
| 194 | MIB-5476 | **M194** | 239 | MIB-5609 | **M239** | 284 | MIB-5716 | **M284** | 329 | MIB-5859 | **M329** |
| 195 | MIB-5477 | **M195** | 240 | MIB-5610 | **M240** | 285 | MIB-5731 | **M285** | 330 | MIB-5864 | **M330** |
| 196 | MIB-5482 | **M196** | 241 | MIB-5611 | **M241** | 286 | MIB-5732 | **M286** | 331 | MIB-5866 | **M331** |
| 197 | MIB-5483 | **M197** | 242 | MIB-5612 | **M242** | 287 | MIB-5735 | **M287** | 332 | MIB-5867 | **M332** |
| 198 | MIB-5484 | **M198** | 243 | MIB-5617 | **M243** | 288 | MIB-5738 | **M288** | 333 | MIB-5872 | **M333** |
| 199 | MIB-5485 | **M199** | 244 | MIB-5621 | **M244** | 289 | MIB-5739 | **M289** | 334 | MIB-5873 | **M334** |
| 200 | MIB-5486 | **M200** | 245 | MIB-5624 | **M245** | 290 | MIB-5745 | **M290** | 335 | MIB-5874 | **M335** |
| 201 | MIB-5487 | **M201** | 246 | MIB-5627 | **M246** | 291 | MIB-5753 | **M291** | 336 | MIB-5876 | **M336** |
| 202 | MIB-5490 | **M202** | 247 | MIB-5631 | **M247** | 292 | MIB-5754 | **M292** | 337 | MIB-5877 | **M337** |
| 203 | MIB-5492 | **M203** | 248 | MIB-5638 | **M248** | 293 | MIB-5755 | **M293** | 338 | MIB-5878 | **M338** |
| 204 | MIB-5495 | **M204** | 249 | MIB-5642 | **M249** | 294 | MIB-5759 | **M294** | 339 | MIB-5883 | **M339** |
| 205 | MIB-5496 | **M205** | 250 | MIB-5644 | **M250** | 295 | MIB-5760 | **M295** | 340 | MIB-5887 | **M340** |
| 206 | MIB-5499 | **M206** | 251 | MIB-5647 | **M251** | 296 | MIB-5766 | **M296** | 341 | MIB-5891 | **M341** |
| 207 | MIB-5502 | **M207** | 252 | MIB-5650 | **M252** | 297 | MIB-5768 | **M297** | 342 | MIB-5893 | **M342** |
| 208 | MIB-5503 | **M208** | 253 | MIB-5654 | **M253** | 298 | MIB-5769 | **M298** | 343 | MIB-5897 | **M343** |
| 209 | MIB-5507 | **M209** | 254 | MIB-5660 | **M254** | 299 | MIB-5770 | **M299** | 344 | MIB-5900 | **M344** |
| 210 | MIB-5511 | **M210** | 255 | MIB-5661 | **M255** | 300 | MIB-5771 | **M300** | 345 | MIB-5906 | **M345** |
| 211 | MIB-5513 | **M211** | 256 | MIB-5662 | **M256** | 301 | MIB-5775 | **M301** | 346 | MIB-5909 | **M346** |
| 212 | MIB-5516 | **M212** | 257 | MIB-5663 | **M257** | 302 | MIB-5776 | **M302** | 347 | MIB-5912 | **M347** |
| 213 | MIB-5518 | **M213** | 258 | MIB-5667 | **M258** | 303 | MIB-5777 | **M303** | 348 | MIB-5917 | **M348** |
| 214 | MIB-5521 | **M214** | 259 | MIB-5669 | **M259** | 304 | MIB-5783 | **M304** | 349 | MIB-5926 | **M349** |
| 215 | MIB-5522 | **M215** | 260 | MIB-5670 | **M260** | 305 | MIB-5786 | **M305** | 350 | MIB-5927 | **M350** |
| 216 | MIB-5526 | **M216** | 261 | MIB-5672 | **M261** | 306 | MIB-5788 | **M306** | 351 | MIB-5928 | **M351** |
| 217 | MIB-5527 | **M217** | 262 | MIB-5674 | **M262** | 307 | MIB-5793 | **M307** | 352 | MIB-5929 | **M352** |
| 218 | MIB-5531 | **M218** | 263 | MIB-5675 | **M263** | 308 | MIB-5794 | **M308** | 353 | MIB-5930 | **M353** |
| 219 | MIB-5532 | **M219** | 264 | MIB-5676 | **M264** | 309 | MIB-5795 | **M309** | 354 | MIB-5932 | **M354** |
| 220 | MIB-5542 | **M220** | 265 | MIB-5678 | **M265** | 310 | MIB-5800 | **M310** | 355 | MIB-5940 | **M355** |
| 221 | MIB-5545 | **M221** | 266 | MIB-5679 | **M266** | 311 | MIB-5802 | **M311** | 356 | MIB-5941 | **M356** |
| 222 | MIB-5546 | **M222** | 267 | MIB-5680 | **M267** | 312 | MIB-5803 | **M312** | 357 | MIB-5944 | **M357** |
| 223 | MIB-5550 | **M223** | 268 | MIB-5681 | **M268** | 313 | MIB-5806 | **M313** | 358 | MIB-5948 | **M358** |
| 224 | MIB-5555 | **M224** | 269 | MIB-5682 | **M269** | 314 | MIB-5809 | **M314** | 359 | MIB-5949 | **M359** |
| 225 | MIB-5556 | **M225** | 270 | MIB-5683 | **M270** | 315 | MIB-5810 | **M315** | 360 | MIB-5953 | **M360** |

**Supplementary Table S1. Continued**

| **S.No.** | **Lines** | **Code** |
| --- | --- | --- |
| 361 | MIB-5955 | **M361** |
| 362 | MIB-5956 | **M362** |
| 363 | MIB-5962 | **M363** |
| 364 | MIB-5963 | **M364** |
| 365 | MIB-5964 | **M365** |
| 366 | MIB-5969 | **M366** |
| 367 | MIB-5971 | **M367** |
| 368 | MIB-5972 | **M368** |
| 369 | MIB-5977 | **M369** |
| 370 | MIB-5981 | **M370** |
| 371 | MIB-5982 | **M371** |
| 372 | MIB-5986 | **M372** |
| 373 | MIB-5992 | **M373** |
| 374 | MIB-5993 | **M374** |
| 375 | MIB-5994 | **M375** |
| 376 | MIB-5995 | **M376** |
| 377 | MIB-5997 | **M377** |
| 378 | MIB-6000 | **M378** |
| 379 | MIB-6001 | **M379** |
| 380 | MIB-6003 | **M380** |
| 381 | MIB-6004 | **M381** |
| 382 | MIB-6012 | **M382** |
| 383 | MIB-6018 | **M383** |
| 384 | MIB-6020 | **M384** |
| 385 | MIB-6021 | **M385** |
| 386 | MIB-6022 | **M386** |
| 387 | MIB-6028 | **M387** |
| 388 | MIB-6033 | **M388** |
| 389 | MIB-6037 | **M389** |
| 390 | MIB-6039 | **M390** |
| 391 | MIB-6040 | **M391** |

**Supplementary Table S1. Continued**

**Supplementary Table S2. Sequence of the 20 designed SNPs**

| **S. No.** | **SNP ID** | **Chr.** | **Allele** | **Sequence** |
| --- | --- | --- | --- | --- |
| 1 | snpOS00912 | 1 | A/C | GCGGCGGCGAGGGAGTTCAACGCGCAGCTGGGCGCGGCGTGCCGGCGGCT[A/C]CGGCAGCGGATGGCGGACGCCGCCGTGGTGTTCACCGACGTGTACGCCGC |
| 2 | snpOS00913 | 1 | C/T | ACAGAGCTCGTCGTCGTCGTCATCGTCATCCAATGTCGGCAGCTGCGAGG[C/T]CGAGCGCGTTCTTGACATTATGGAGCGCGGTGGTGGTGGTGGCAGCAGTG |
| 3 | snpOS00914 | 1 | C/A | AGCAGTACGCCGCGCGCCCGCGCCGCTGTGCGTGCACGGCGTCGGTATGT[C/A]CGCGTGCGTTGGCTGATGCAAGCTCGTCGTAGGCACGTAACGTAATGCGG |
| 4 | snpOS00915 | 1 | T/G | ACGCCATTTCTAATCACGTCCATACGGGGGACGCTGCTCATAACAGCGTC[C/T]AGACTCTGGTAAAGCCGTGACTGCCCGTTGGTCCAATCCACCGCACGCGC |
| 5 | snpOS00916 | 1 | A/T | AGTGGGCGCCGCAGGCGCAGCGCCAGAGGCGGCCGCAGTTCTTCTCGTGG[T/G]TGCGCCAGTCGCCGCGGACGGCGAAGCGCTTGGCGCAGCGGCGGCAGGCG |
| 6 | snpOS00917 | 1 | C/T | GTTTATCATCGTCTTGTTCACGTGCAGCGCGGCCAGGAAGAAGAAGCAAG[T/C]TCGCGCGAAGAACGGCGACACGCCTGAGCCTGACGCCGCCGGCGGTGCCC |
| 7 | snpOS00918 | 1 | T/G | TCCCGGCCACGTCACCCGCCGCCGCCGACGAGGTGAATTCCGCTGGCCTA[T/G]TGTCAGCGTCCATGGTCGGTTCTTGGTAGCTCCATGGCTGCTGCTGCTCG |
| 8 | snpOS00919 | 2 | T/C | AAACCATGTCAGATGGTTGGGTAGCTTAATTAGTAGAGTATAGTTAATTA(A/G)GGTTTGTATCGCTTTTTAGCTGCTTAAATTCTTACCCCTGTTTATATTTA |
| 9 | snpOS00920 | 5 | T/G | CAGGCAGATCAGGGGTGCAGAGGCACCATTTAATCGATCGGCCATTCCTT[T/C]CGATATGCTGCTGCTGCTGCTGCACGTCTTGCTTCTTCATTCCCAGCGAG |
| 10 | snpOS00921 | 5 | T/C | CGCTGCTGCTGCTACTACTACCGCCTCCGCCGCTCCCCACAGCCGCCTCC[G/A]CGCTGCCGACGCCGCCCTTGGAGCAGTAGAACCGGGACGACGGCCGCCGC |
| 11 | snpOS00922 | 6 | T/C | TTCGACGAGATGCCCCAGAGGAACGCCGCTTCCTGGAACACGATGGTCAC[T/C]GGGTTCTTTGCTGCTGGCCAGGTGGTGAAGGCCCTTGACGTGTTCGATGC |
| 12 | snpOS00923 | 6 | G/A | CAGCTCGCCTCCGCGCTCGCGGCGGCCGCGGCCGTCGAGGCGTCCGCCCC[A/C]AGCGCGGACCTCGCCGCCTACCTCCAGGCCCTCCTCCCGCGCCTCCTCAA |
| 13 | snpOS00924 | 6 | T/C | CCCCACCGCCGCCACCACATCACCGGCTCCAACGACTCCATGACTACCAC[G/A]ATACCGCGGCCACCACCACCTCGCTCCACGACCACCTCGTCACTGCAGCC |
| 14 | snpOS00925 | 6 | A/C | AATGAATTAGGCAAGAAATAAAAAGCAGCAAGCAACAAAGCACTGTAGAT(C/T)TCAATTTTTAGATGTGTTTTGTTGATCTGAAGATAGACACTAGAAGTGAG |
| 15 | snpOS00926 | 6 | G/A | ATCATCCTGTTAGCCGTCCAGCAGCGGTGTTTCAATTAATGCCAATCAAA[G/A]AAAGAGCTTGCATTTGATTGGCCCACCGAGTTGGTTCGTGCAAAGGAAGG |
| 16 | snpOS00927 | 6 | A/G | GAACGCTACATGATGCAGCCACTAAACTCCCAAAGCTTTCTTGACAGTGG[A/T]GGTGGTCTCGGCAGCTGCCTGCCTGCCTGCTCCACTGATGCAGCTGAGCT |
| 17 | snpOS00928 | 6 | A/T | TACATCTGCTGCTCTGGCTGTTGCTGAGGGGCATGCTGCATGTCCCAGCC[G/A]TTGAAGTAAGGCCGGCCATGGCGGACATGGTGAACTTTTTCAGGCTCCTT |
| 18 | snpOS00929 | 6 | G/A | GGGCAGCGCGCAGGGGCAGGGAGGTGGGCAGCGGCGCGGGGACGGGCGGT[A/G]AGCTGGAGGGCGGTGGAGGATGCCGCCGCGCCACGGCTGCACGCCGTGCG |
| 19 | snpOS00930 | 6 | G/A | AGGCCGCAGCTTAAAGGCAGCCATCGCTCTAATCTGCTATGGTGACGACG[G/A]CAGCGGCTGCTGCTGCTTCTGTAAGGGCCCTGCTGCTCCCTTGTGGCAGC |
| 20 | snpOS00931 | 7 | C/A | GGAGTGAGTGGGGCGGAGACCCAGGCTGAACCGCCATCAATGTGCAGCGC[C/A]CCAACCATCCCAATGCGGCGAGGGCGGACGTGCAGCGCTGCCCGATTGAC |

**Supplementary Table S3.** **Reaction of rice genotypes to Brown Planthopper**

| **S.No.** | **Genotype** | **Damage score** | | | | | | |
| --- | --- | --- | --- | --- | --- | --- | --- | --- |
|  |  | ***Kharif* 2018** | | | ***Rabi* 2018-19** | | | **Overall mean** |
|  |  | **Trial I** | **Trial II** | **Mean** | **Trial I** | **Trial II** | **Mean** |  |
| 1 | **PTB33** | 1.5 | 1.3 | 1.4 | 1.4 | 1.6 | 1.5 | 1.5 |
| 2 | **RathuHeenati** | 1.5 | 1.7 | 1.6 | 1.8 | 1.7 | 1.8 | 1.7 |
| 3 | **RP2068-18-3-5** | 1.8 | 2.1 | 2.0 | 1.8 | 1.7 | 1.8 | 1.9 |
| 4 | **TN1** | 9.0 | 9.0 | 9.0 | 9.0 | 9.0 | 9.0 | 9.0 |
| 5 | **BPT5204** | 9.0 | 9.0 | 9.0 | 9.0 | 9.0 | 9.0 | 9.0 |
| 6 | Mudgo | 3.3 | 3.0 | 3.2 | 3.6 | 3.5 | 3.6 | 3.4 |
| 7 | IR 64 | 6.0 | 7.0 | 6.5 | 6.5 | 7.3 | 6.9 | 6.7 |
| 8 | ASD 7 | 8.9 | 9.0 | 9.0 | 8.6 | 8.9 | 8.8 | 8.9 |
| 9 | Milyang 63 | 9.0 | 9.0 | 9.0 | 9.0 | 9.0 | 9.0 | 9.0 |
| 10 | RathuHeenati | 2.5 | 1.9 | 2.2 | 2.2 | 2.3 | 2.3 | 2.2 |
| 11 | Babawee | 8.2 | 8.6 | 8.4 | 8.3 | 8.6 | 8.5 | 8.4 |
| 12 | ARC 10550 | 9.0 | 9.0 | 9.0 | 9.0 | 9.0 | 9.0 | 9.0 |
| 13 | Swarnalatha | 2.7 | 4.5 | 3.6 | 4.4 | 3.6 | 4.0 | 3.8 |
| 14 | T12 | 4.7 | 5.2 | 5.0 | 5.1 | 4.9 | 5.0 | 5.0 |
| 15 | Chinsaba | 9.0 | 9.0 | 9.0 | 9.0 | 9.0 | 9.0 | 9.0 |
| 16 | Pokkali | 9.0 | 9.0 | 9.0 | 9.0 | 9.0 | 9.0 | 9.0 |
| 17 | IR65482-7-216 | 7.4 | 7.3 | 7.4 | 7.5 | 7.1 | 7.3 | 7.3 |
| 18 | IR71033-121-15 | 8.8 | 8.7 | 8.8 | 8.6 | 8.5 | 8.6 | 8.7 |
| 19 | MUT NS1 | 9.0 | 9.0 | 9.0 | 9.0 | 9.0 | 9.0 | 9.0 |
| 20 | OM 4498 | 6.9 | 7.2 | 7.1 | 6.8 | 6.8 | 6.8 | 6.9 |
| 21 | RP2068-18-3-5 | 3.5 | 3.7 | 3.6 | 2.2 | 2.0 | 2.1 | 2.9 |
| 22 | MO1 | 7.6 | 8.1 | 7.9 | 8.0 | 7.6 | 7.8 | 7.8 |
| 23 | MTU 1010 | 7.9 | 8.4 | 8.2 | 8.6 | 8.7 | 8.7 | 8.4 |
| 24 | RP BIO 4918-230S | 1.7 | 2.3 | 2.0 | 2.1 | 1.9 | 2.0 | 2.0 |
| 25 | IR 26 | 8.6 | 8.7 | 8.7 | 8.6 | 8.3 | 8.5 | 8.6 |
|  | PTB33 | 1.6 | 1.6 | 1.6 | 1.7 | 1.8 | 1.8 | 1.7 |
|  | RathuHeenati | 1.7 | 1.8 | 1.8 | 1.9 | 1.7 | 1.8 | 1.8 |
|  | RP2068-18-3-5 | 1.9 | 2.1 | 2.0 | 1.8 | 2.0 | 1.9 | 2.0 |
|  | TN1 | 9.0 | 9.0 | 9.0 | 9.0 | 9.0 | 9.0 | 9.0 |
|  | BPT5204 | 9.0 | 9.0 | 9.0 | 9.0 | 9.0 | 9.0 | 9.0 |
| 26 | IR 40 | 6.9 | 6.8 | 6.9 | 7.4 | 7.2 | 7.3 | 7.1 |
| 27 | IR 66 | 8.5 | 7.9 | 8.2 | 8.5 | 7.8 | 8.2 | 8.2 |
| 28 | IR 72 | 5.0 | 5.6 | 5.3 | 4.9 | 5.7 | 5.3 | 5.3 |
| 29 | Utrirajappan | 9.0 | 9.0 | 9.0 | 9.0 | 9.0 | 9.0 | 9.0 |
| 30 | Ndiang Marie | 7.9 | 7.6 | 7.8 | 8.1 | 7.9 | 8.0 | 7.9 |
| 31 | Sinna Sivappu | 5.0 | 4.9 | 5.0 | 5.0 | 4.9 | 5.0 | 5.0 |
| 32 | Balamwee | 9.0 | 9.0 | 9.0 | 9.0 | 9.0 | 9.0 | 9.0 |
| 33 | IR 62 | 7.8 | 7.9 | 7.9 | 7.6 | 7.8 | 7.7 | 7.8 |
| 34 | RathuHeenati accession | 3.7 | 3.6 | 3.7 | 3.9 | 3.7 | 3.8 | 3.7 |
| 35 | IR65482-136-2-2 | 7.8 | 9.0 | 8.4 | 7.9 | 8.9 | 8.4 | 8.4 |
| 36 | M1 | 6.5 | 8.5 | 7.5 | 8.1 | 8.1 | 8.1 | 7.8 |
| 37 | M4 | 1.3 | 2.2 | 1.7 | 2.4 | 2.5 | 2.5 | 2.1 |
| 38 | M61 | 3.1 | 5.0 | 4.1 | 4.3 | 4.5 | 4.4 | 4.2 |
| 39 | M80 | 5.5 | 6.1 | 5.8 | 5.7 | 6.2 | 6.0 | 5.9 |
| 40 | M88 | 2.3 | 2.1 | 2.2 | 0.8 | 2.2 | 1.5 | 1.9 |
| 41 | M123 | 8.4 | 6.2 | 7.3 | 7.4 | 7.7 | 7.6 | 7.4 |
| 42 | M131 | 9.0 | 8.3 | 8.7 | 8.6 | 8.4 | 8.5 | 8.6 |
| 43 | M179 | 3.0 | 3.1 | 3.1 | 2.1 | 1.4 | 1.8 | 2.4 |
| 44 | M182 | 2.7 | 2.9 | 2.8 | 1.9 | 3.2 | 2.6 | 2.7 |
| 45 | M187 | 3.8 | 3.1 | 3.5 | 3.4 | 3.9 | 3.7 | 3.6 |
|  | **PTB33** | 1.9 | 1.9 | 1.9 | 1.4 | 1.8 | 1.6 | 1.8 |
|  | **RathuHeenati** | 1.6 | 1.8 | 1.7 | 1.9 | 2.0 | 2.0 | 1.8 |
|  | **RP2068-18-3-5** | 2.0 | 2.2 | 2.1 | 1.8 | 1.9 | 1.9 | 2.0 |
|  | **TN1** | 9.0 | 9.0 | 9.0 | 9.0 | 9.0 | 9.0 | 9.0 |
|  | **BPT5204** | 9.0 | 9.0 | 9.0 | 9.0 | 9.0 | 9.0 | 9.0 |
| 46 | M189 | 7.6 | 8.3 | 7.9 | 7.8 | 7.9 | 7.9 | 7.9 |
| 47 | M190 | 9.0 | 7.8 | 8.4 | 8.8 | 7.9 | 8.4 | 8.4 |
| 48 | M192 | 2.1 | 3.5 | 2.8 | 3.4 | 2.8 | 3.1 | 3.0 |
| 49 | M201 | 3.2 | 3.0 | 3.1 | 3.5 | 3.7 | 3.6 | 3.4 |
| 50 | M227 | 7.1 | 8.2 | 7.7 | 8.7 | 9.0 | 8.9 | 8.3 |
| 51 | M229 | 2.9 | 3.0 | 3.0 | 3.2 | 3.0 | 3.1 | 3.0 |
| 52 | M240 | 2.8 | 3.1 | 3.0 | 0.5 | 2.3 | 1.4 | 2.2 |
| 53 | M262 | 7.7 | 7.6 | 7.7 | 7.6 | 7.9 | 7.8 | 7.7 |
| 54 | M267 | 7.0 | 7.0 | 7.0 | 6.9 | 7.5 | 7.2 | 7.1 |
| 55 | M272 | 9.0 | 9.0 | 9.0 | 9.0 | 9.0 | 9.0 | 9.0 |
| 56 | M276 | 3.4 | 3.9 | 3.7 | 1.0 | 4.7 | 2.9 | 3.3 |
| 57 | M278 | 5.5 | 5.8 | 5.6 | 6.1 | 6.3 | 6.2 | 5.9 |
| 58 | M279 | 5.0 | 6.2 | 5.6 | 5.5 | 5.6 | 5.6 | 5.6 |
| 59 | M284 | 2.1 | 4.4 | 3.3 | 3.9 | 4.1 | 4.0 | 3.6 |
| 60 | M286 | 2.7 | 3.9 | 3.3 | 4.0 | 4.3 | 4.2 | 3.7 |
| 61 | M289 | 6.2 | 7.5 | 6.8 | 6.2 | 6.9 | 6.6 | 6.7 |
| 62 | M293 | 6.3 | 6.1 | 6.2 | 5.4 | 5.8 | 5.6 | 5.9 |
| 63 | M296 | 5.5 | 6.4 | 6.0 | 5.9 | 5.7 | 5.8 | 5.9 |
| 64 | M304 | 9.0 | 9.0 | 9.0 | 9.0 | 9.0 | 9.0 | 9.0 |
| 65 | M306 | 7.4 | 7.8 | 7.6 | 8.6 | 8.1 | 8.4 | 8.0 |
|  | **PTB33** | 1.5 | 1.6 | 1.6 | 1.4 | 1.6 | 1.5 | 1.5 |
|  | **RathuHeenati** | 1.6 | 1.5 | 1.6 | 1.6 | 1.8 | 1.7 | 1.6 |
|  | **RP2068-18-3-5** | 1.9 | 2.2 | 2.1 | 1.7 | 1.8 | 1.8 | 1.9 |
|  | **TN1** | 9.0 | 9.0 | 9.0 | 9.0 | 9.0 | 9.0 | 9.0 |
|  | **BPT5204** | 9.0 | 9.0 | 9.0 | 9.0 | 9.0 | 9.0 | 9.0 |
| 66 | M312 | 2.3 | 3.2 | 2.8 | 3.3 | 3.1 | 3.2 | 3.0 |
| 67 | M344 | 1.8 | 3.3 | 2.6 | 3.1 | 2.1 | 2.6 | 2.6 |
| 68 | M359 | 2.2 | 3.2 | 2.7 | 3.8 | 4.1 | 4.0 | 3.3 |
| 69 | M362 | 7.3 | 6.7 | 7.0 | 7.4 | 7.4 | 7.4 | 7.2 |
| 70 | M364 | 7.4 | 8.0 | 7.7 | 7.6 | 7.8 | 7.7 | 7.7 |
| 71 | M384 | 6.6 | 6.4 | 6.5 | 6.9 | 6.7 | 6.8 | 6.7 |
| 72 | IET23993 | 4.7 | 5.5 | 5.1 | 4.9 | 4.8 | 4.9 | 5.0 |
| 73 | BM71 | 2.1 | 4.3 | 3.2 | 3.4 | 4.1 | 3.7 | 3.5 |
| 74 | RPV1355 | 3.7 | 3.6 | 3.7 | 2.0 | 4.0 | 3.0 | 3.3 |
| 75 | KNM118 | 9.0 | 9.0 | 9.0 | 8.7 | 8.8 | 8.8 | 8.9 |
| 76 | 10-3 | 2.4 | 3.0 | 2.7 | 3.1 | 2.4 | 2.8 | 2.7 |
| 77 | Telangana Sona | 9.0 | 9.0 | 9.0 | 9.0 | 9.0 | 9.0 | 9.0 |
| 78 | 3K-19 | 6.9 | 7.6 | 7.3 | 7.2 | 6.8 | 7.0 | 7.1 |
| 79 | 3K-47 | 9.0 | 9.0 | 9.0 | 9.0 | 8.9 | 9.0 | 9.0 |
| 80 | 3K-53 | 7.9 | 9.0 | 8.5 | 9.0 | 9.0 | 9.0 | 8.7 |
| 81 | 3K-59 | 4.9 | 5.5 | 5.2 | 5.4 | 5.1 | 5.3 | 5.2 |
| 82 | 3K-132 | 8.8 | 9.0 | 8.9 | 8.7 | 8.6 | 8.7 | 8.8 |
| 83 | 3K-168 | 8.1 | 9.0 | 8.6 | 7.9 | 8.4 | 8.2 | 8.4 |
| 84 | 3K-187 | 8.1 | 9.0 | 8.6 | 8.6 | 8.1 | 8.4 | 8.5 |
| 85 | 3K-200 | 7.7 | 9.0 | 8.4 | 7.8 | 7.9 | 7.9 | 8.1 |
|  | **PTB33** | 1.8 | 1.3 | 1.6 | 1.7 | 1.6 | 1.7 | 1.6 |
|  | **RathuHeenati** | 1.6 | 1.7 | 1.7 | 1.8 | 1.9 | 1.9 | 1.8 |
|  | **RP2068-18-3-5** | 1.9 | 2.1 | 2.0 | 1.7 | 1.7 | 1.7 | 1.9 |
|  | **TN1** | 9.0 | 9.0 | 9.0 | 9.0 | 9.0 | 9.0 | 9.0 |
|  | **BPT5204** | 9.0 | 9.0 | 9.0 | 9.0 | 9.0 | 9.0 | 9.0 |
| 86 | 3K-202 | 5.9 | 7.1 | 6.5 | 6.1 | 6.6 | 6.4 | 6.4 |
| 87 | 3K-290 | 5.9 | 8.2 | 7.1 | 7.5 | 7.4 | 7.5 | 7.3 |
| 88 | 3K-322 | 8.6 | 9.0 | 8.8 | 8.5 | 8.4 | 8.5 | 8.6 |

**Supplementary Table S4. Twenty-three significant SNPs identified from GLM model**

| **S.NO.** | **Gene Locus ID** | **Chromosome** | **SNPs** | **Putative function** |
| --- | --- | --- | --- | --- |
| 1 | [LOC_Os01g22640.1](http://rice.plantbiology.msu.edu/cgi-bin/ORF_infopage.cgi?orf=LOC_Os01g22640.1) | 1 | S1_12737403, S1_12742211 | GDSL-like lipase/acylhydrolase, putative, expressed |
| 2 | [LOC_Os01g23610.1](http://rice.plantbiology.msu.edu/cgi-bin/ORF_infopage.cgi?orf=LOC_Os01g23610.1) | 1 | S1_13264894, S1_13274423 | Dihydrolipoyl dehydrogenase, putative, expressed |
| 3 | [LOC_Os01g23680.1](http://rice.plantbiology.msu.edu/cgi-bin/ORF_infopage.cgi?orf=LOC_Os01g23680.1) | 1 | S1_13316461, S1_13323794 | Rossmann fold nucleotide-binding protein involved in DNA uptake, putative, expressed |
| 4 | [LOC_Os01g23770.1](http://rice.plantbiology.msu.edu/cgi-bin/ORF_infopage.cgi?orf=LOC_Os01g23770.1) | 1 | S1_13333531, S1_13365703 | OsMADS93-MADS-box family gene with M-beta type-box, expressed |
| 5 | [LOC_Os01g24050.1](http://rice.plantbiology.msu.edu/cgi-bin/ORF_infopage.cgi?orf=LOC_Os01g24050.1) | 1 | S1_13541645, S1_13563387 | Expressed protein |
| 6 | [LOC_Os01g24950.1](http://rice.plantbiology.msu.edu/cgi-bin/ORF_infopage.cgi?orf=LOC_Os01g24950.1) | 1 | S1_14060399 | Exocyst complex component 6, putative, expressed |
| 7 | [LOC_Os02g10240.1](http://rice.plantbiology.msu.edu/cgi-bin/ORF_infopage.cgi?orf=LOC_Os02g10240.1) | 2 | S2_5364800 | ZOS2-05-C2H2 zinc finger protein, expressed |
| 8 | [LOC_Os06g07420.1](http://rice.plantbiology.msu.edu/cgi-bin/ORF_infopage.cgi?orf=LOC_Os06g07420.1) | 6 | S6_3566382 | Retrotransposon protein, putative, unclassified, expressed |
| 9 | [LOC_Os06g07620.1](http://rice.plantbiology.msu.edu/cgi-bin/ORF_infopage.cgi?orf=LOC_Os06g07620.1) | 6 | S6_3682423 | Expressed protein |
| 10 | [LOC_Os06g15730.1](http://rice.plantbiology.msu.edu/cgi-bin/ORF_infopage.cgi?orf=LOC_Os06g15730.1)  [LOC_Os06g15740.1](http://rice.plantbiology.msu.edu/cgi-bin/ORF_infopage.cgi?orf=LOC_Os06g15730.1) | 6 | S6_8914643, S6_8914650, S6_8914651, S6_8914671, S6_8921200, S6_8921201 | Peroxidase precursor, Pentatricopeptide repeat-containing protein, expressed |
| 11 | LOC_Os06g15810.1 | 6 | S6_8975500 | Integral membrane protein, putative, expressed |
| 12 | [LOC_Os06g15850.1](http://rice.plantbiology.msu.edu/cgi-bin/ORF_infopage.cgi?orf=LOC_Os06g15850.1) | 6 | S6_9003866, S6_9004225 | Nucleotide binding, response to stress, expressed |

**Supplementary Table S5. Validation of the 20 designed SNPs in the genotypes**

| **S.No.** | **Genotype** | **snpOS00912** | **snpOS00913** | **snpOS00914** | **snpOS00915** | **snpOS00916** |
| --- | --- | --- | --- | --- | --- | --- |
| 1 | Mudgo | A:A | T:T | A:A | C:C | G:G |
| 2 | IR 64 | A:A | T:T | A:A | T:T | G:G |
| 3 | ASD 7 | A:A | T:T | A:A | C:C | G:G |
| 4 | Milyang 63 | ? | T:T | A:A | C:C | G:G |
| 5 | RathuHeenati | A:A | T:T | A:A | C:C | G:G |
| 6 | Babawee | A:A | T:T | A:A | T:T | G:G |
| 7 | ARC 10550 | ? | T:T | A:A | C:C | G:G |
| 8 | Swarnalatha | Uncallable | T:T | A:A | C:C | G:G |
| 9 | T12 | C:A | T:T | A:A | C:C | G:G |
| 10 | Chinsaba | A:A | T:T | A:A | C:C | G:G |
| 11 | Pokkali | C:A | T:T | A:A | C:C | G:G |
| 12 | IR65482-7-216 | A:A | T:T | A:A | C:C | G:G |
| 13 | IR71033-121-15 | A:A | T:T | A:A | C:C | G:G |
| 14 | MUT NS1 | A:A | T:T | ? | T:T | G:G |
| 15 | OM 4498 | C:A | T:T | A:A | C:C | G:G |
| 16 | RP2068-18-3-5 | C:A | T:T | A:A | C:C | G:G |
| 17 | MO1 | A:A | T:T | A:A | C:C | G:G |
| 18 | MTU 1010 | A:A | T:T | A:A | C:C | G:G |
| 19 | RP BIO 4918-230S | A:A | T:T | A:A | C:C | G:G |
| 20 | IR 26 | A:A | T:T | A:A | ? | G:G |
| 21 | IR 40 | A:A | T:T | A:A | T:C | G:G |
| 22 | IR 66 | A:A | T:T | A:A | C:C | G:G |
| 23 | IR 72 | A:A | T:T | A:A | T:T | G:G |
| 24 | Utrirajappan | A:A | T:T | A:A | C:C | G:G |
| 25 | Ndiang Marie | C:A | T:T | A:A | C:C | G:G |
| 26 | Sinna Sivappu | C:A | T:T | A:A | C:C | G:G |
| 27 | Balamwee | A:A | T:T | A:A | C:C | G:G |
| 28 | IR 62 | A:A | T:T | A:A | T:C | G:G |
| 29 | RathuHeenati accession | A:A | T:T | A:A | C:C | G:G |
| 30 | IR65482-136-2-2 | C:A | T:T | A:A | C:C | G:G |
| 31 | M1 | A:A | T:T | A:A | C:C | G:G |
| 32 | M4 | A:A | T:T | A:A | T:C | G:G |
| 33 | M61 | C:A | T:T | A:A | C:C | G:G |
| 34 | M80 | Uncallable | T:T | A:A | C:C | G:G |
| 35 | M88 | A:A | T:T | ? | T:T | G:G |
| 36 | M123 | A:A | T:T | A:A | C:C | G:G |
| 37 | M131 | A:A | T:T | A:A | C:C | G:G |
| 38 | M179 | A:A | T:T | A:A | C:C | G:G |
| 39 | M182 | A:A | T:T | A:A | T:T | G:G |
| 40 | M187 | A:A | T:T | A:A | ? | ? |
| 41 | M189 | C:C | T:T | A:A | C:C | G:G |
| 42 | M190 | C:C | ? | A:A | ? | G:G |
| 43 | M192 | C:A | T:T | A:A | C:C | G:G |
| 44 | M201 | A:A | T:T | A:A | T:T | G:G |
| 45 | M227 | A:A | T:T | A:A | T:T | G:G |

| **S.No.** | **Genotype** | **snpOS00912** | **snpOS00913** | **snpOS00914** | **snpOS00915** | **snpOS00916** |
| --- | --- | --- | --- | --- | --- | --- |
| 46 | M240 | C:A | T:T | A:A | T:T | G:G |
| 47 | M262 | A:A | T:T | A:A | T:T | G:G |
| 48 | M267 | A:A | T:T | A:A | T:T | G:G |
| 49 | M272 | A:A | T:T | A:A | C:C | G:G |
| 50 | M276 | A:A | T:T | A:A | T:C | G:G |
| 51 | M278 | C:A | T:T | A:A | C:C | G:G |
| 52 | M279 | A:A | T:T | A:A | C:C | G:G |
| 53 | M284 | C:A | T:T | A:A | C:C | G:G |
| 54 | M286 | C:A | T:T | A:A | C:C | G:G |
| 55 | M289 | C:A | T:T | A:A | C:C | G:G |
| 56 | M293 | C:A | T:T | A:A | C:C | G:G |
| 57 | M296 | A:A | T:T | A:A | C:C | G:G |
| 58 | M304 | A:A | T:T | A:A | T:C | G:G |
| 59 | M306 | A:A | T:T | A:A | C:C | G:G |
| 60 | M312 | C:A | T:T | A:A | C:C | G:G |
| 61 | M359 | C:A | T:T | A:A | C:C | G:G |
| 62 | M344 | A:A | T:T | A:A | C:C | G:G |
| 63 | M362 | C:A | T:T | A:A | C:C | G:G |
| 64 | M364 | A:A | T:T | A:A | T:T | G:G |
| 65 | M384 | A:A | T:T | A:A | C:C | G:G |
| 66 | IET23993 | A:A | T:T | A:A | T:T | G:G |
| 67 | BM71 | A:A | T:T | A:A | T:T | G:G |
| 68 | RPV1355 | Uncallable | T:T | ? | T:T | G:G |
| 69 | KNM118 | C:A | T:T | A:A | C:C | G:G |
| 70 | 10-3 | A:A | T:T | A:A | C:C | G:G |
| 71 | Telangana Sona | C:A | T:T | A:A | T:T | G:G |
| 72 | M229 | A:A | T:T | A:A | C:C | G:G |
| 73 | 3K-19 | C:A | T:T | A:A | C:C | G:G |
| 74 | 3K-47 | C:A | T:T | A:A | C:C | G:G |
| 75 | 3K-53 | A:A | T:T | A:A | C:C | G:G |
| 76 | 3K-59 | A:A | T:T | A:A | C:C | G:G |
| 77 | 3K-132 | A:A | T:T | A:A | C:C | G:G |
| 78 | 3K-168 | C:A | T:T | A:A | C:C | G:G |
| 79 | 3K-187 | A:A | T:T | A:A | C:C | G:G |
| 80 | 3K-200 | A:A | T:T | A:A | C:C | G:G |
| 81 | 3K-202 | A:A | T:T | A:A | C:C | G:G |
| 82 | 3K-290 | A:A | T:T | A:A | C:C | G:G |
| 83 | 3K-322 | A:A | T:T | A:A | C:C | G:G |
| 84 | PTB33 | A:A | T:T | A:A | C:C | G:G |
| 85 | RathuHeenati | A:A | T:T | A:A | C:C | G:G |
| 86 | RP2068-18-3-5 | C:A | T:T | A:A | C:C | G:G |
| 87 | TN1 | ? | T:T | A:A | C:C | ? |
| 88 | BPT5204 | A:A | T:T | A:A | C:C | G:G |

| **S.No.** | **Genotype** | **snpOS00917** | **snpOS00918** | **snpOS00919** | **snpOS00920** | **snpOS00921** |
| --- | --- | --- | --- | --- | --- | --- |
| 1 | Mudgo | T:T | T:T | A:A | C:C | G:G |
| 2 | IR 64 | T:T | T:T | A:A | C:T | A:G |
| 3 | ASD 7 | T:T | T:T | A:A | C:C | G:G |
| 4 | Milyang 63 | T:T | T:T | A:A | C:C | G:G |
| 5 | RathuHeenati | T:T | T:T | A:A | T:T | G:G |
| 6 | Babawee | T:T | T:T | A:A | T:T | G:G |
| 7 | ARC 10550 | T:T | T:T | A:A | T:T | A:A |
| 8 | Swarnalatha | T:T | T:T | A:A | T:T | A:A |
| 9 | T12 | T:T | T:T | A:A | T:T | G:G |
| 10 | Chinsaba | T:T | T:T | A:A | C:C | G:G |
| 11 | Pokkali | T:T | T:T | A:A | C:C | A:A |
| 12 | IR65482-7-216 | T:T | T:T | A:A | C:C | A:A |
| 13 | IR71033-121-15 | T:T | T:T | A:A | C:C | A:A |
| 14 | MUT NS1 | T:T | T:T | A:A | T:T | G:G |
| 15 | OM 4498 | T:T | T:T | A:A | C:C | A:A |
| 16 | RP 2068-18-3-5 | T:T | T:T | A:A | C:C | A:A |
| 17 | MO1 | T:T | T:T | A:A | C:C | G:G |
| 18 | MTU 1010 | T:T | T:T | A:A | T:T | G:G |
| 19 | RP BIO 4918-230S | T:T | T:T | A:A | C:C | G:G |
| 20 | IR 26 | T:T | T:T | A:A | C:C | A:A |
| 21 | IR 40 | T:T | T:T | A:A | C:C | G:G |
| 22 | IR 66 | T:T | T:T | A:A | T:T | G:G |
| 23 | IR 72 | T:T | T:T | A:A | T:T | G:G |
| 24 | Utrirajappan | T:T | T:T | A:A | T:T | G:G |
| 25 | Ndiang Marie | T:T | T:T | A:A | T:T | G:G |
| 26 | Sinna Sivappu | T:T | T:T | A:A | C:T | A:G |
| 27 | Balamwee | T:T | T:T | A:A | T:T | G:G |
| 28 | IR 62 | T:T | T:T | A:A | T:T | G:G |
| 29 | RathuHeenati accession | T:T | T:T | A:A | ? | G:G |
| 30 | IR65482-136-2-2 | T:T | T:T | A:A | C:C | G:G |
| 31 | M1 | T:T | T:T | A:A | T:T | G:G |
| 32 | M4 | T:T | T:T | A:A | C:T | A:G |
| 33 | M61 | T:T | T:T | A:A | T:T | G:G |
| 34 | M80 | C:C | G:G | G:G | T:T | G:G |
| 35 | M88 | C:C | G:G | G:G | T:T | G:G |
| 36 | M123 | T:T | T:T | A:A | T:T | G:G |
| 37 | M131 | T:T | T:T | A:A | T:T | G:G |
| 38 | M179 | C:C | G:G | G:G | T:T | G:G |
| 39 | M182 | C:C | G:G | G:G | T:T | G:G |
| 40 | M187 | T:T | T:T | A:A | C:C | A:A |
| 41 | M189 | ? | ? | A:A | ? | A:A |
| 42 | M190 | ? | ? | ? | T:T | A:G |
| 43 | M192 | T:T | T:T | A:A | T:T | G:G |
| 44 | M201 | T:T | T:T | A:A | T:T | G:G |
| 45 | M227 | T:T | T:T | A:A | C:C | G:G |

| **S.No.** | **Genotype** | **snpOS00917** | **snpOS00918** | **snpOS00919** | **snpOS00920** | **snpOS00921** |
| --- | --- | --- | --- | --- | --- | --- |
| 46 | M240 | T:T | T:T | A:A | T:T | G:G |
| 47 | M262 | T:T | T:T | A:A | T:T | G:G |
| 48 | M267 | T:T | T:T | A:A | T:T | G:G |
| 49 | M272 | T:T | T:T | A:A | T:T | G:G |
| 50 | M276 | T:T | T:T | A:A | T:T | G:G |
| 51 | M278 | T:T | T:T | A:A | C:C | A:A |
| 52 | M279 | T:T | T:T | A:A | T:T | G:G |
| 53 | M284 | T:T | T:T | A:A | T:T | G:G |
| 54 | M286 | T:T | T:T | A:A | T:T | G:G |
| 55 | M289 | T:T | T:T | A:A | C:C | A:A |
| 56 | M293 | C:C | G:G | G:G | T:T | G:G |
| 57 | M296 | T:T | T:T | A:A | C:C | A:A |
| 58 | M304 | T:T | T:T | A:A | C:T | A:G |
| 59 | M306 | T:T | T:T | A:A | T:T | G:G |
| 60 | M312 | T:T | T:T | A:A | C:C | A:A |
| 61 | M359 | T:T | T:T | A:A | T:T | G:G |
| 62 | M344 | C:T | G:T | G:A | T:T | G:G |
| 63 | M362 | T:T | T:T | A:A | T:T | G:G |
| 64 | M364 | T:T | T:T | A:A | C:C | A:A |
| 65 | M384 | T:T | T:T | A:A | T:T | G:G |
| 66 | IET23993 | T:T | T:T | A:A | C:C | G:G |
| 67 | BM71 | C:C | G:G | G:G | T:T | G:G |
| 68 | RPV1355 | T:T | T:T | A:A | C:C | G:G |
| 69 | KNM118 | T:T | T:T | A:A | T:T | G:G |
| 70 | 10-3 | T:T | T:T | A:A | C:C | A:A |
| 71 | Telangana Sona | T:T | T:T | A:A | T:T | A:A |
| 72 | M229 | T:T | T:T | A:A | T:T | G:G |
| 73 | 3K-19 | T:T | T:T | A:A | C:C | G:G |
| 74 | 3K-47 | T:T | T:T | A:A | C:T | A:G |
| 75 | 3K-53 | T:T | T:T | A:A | T:T | G:G |
| 76 | 3K-59 | T:T | T:T | A:A | T:T | G:G |
| 77 | 3K-132 | T:T | T:T | A:A | T:T | G:G |
| 78 | 3K-168 | T:T | T:T | A:A | T:T | A:A |
| 79 | 3K-187 | T:T | T:T | A:A | C:C | G:G |
| 80 | 3K-200 | T:T | T:T | A:A | C:C | G:G |
| 81 | 3K-202 | T:T | T:T | A:A | T:T | G:G |
| 82 | 3K-290 | T:T | T:T | A:A | C:C | G:G |
| 83 | 3K-322 | C:T | G:T | G:A | C:T | A:G |
| 84 | PTB33 | T:T | T:T | A:A | T:T | G:G |
| 85 | RathuHeenati | T:T | T:T | A:A | T:T | G:G |
| 86 | RP2068-18-3-5 | T:T | T:T | A:A | C:C | A:A |
| 87 | TN1 | T:T | T:T | A:A | ? | ? |
| 88 | BPT5204 | T:T | T:T | A:A | T:T | G:G |
| **S.No.** | **Genotype** | **snpOS00922** | **snpOS00923** | **snpOS00924** | **snpOS00925** | **snpOS00926** |
| 1 | Mudgo | T:T | A:A | G:G | C:C | G:G |
| 2 | IR 64 | C:T | C:A | G:G | C:C | A:G |
| 3 | ASD 7 | T:T | A:A | A:A | C:C | G:G |
| 4 | Milyang 63 | T:T | A:A | A:G | C:C | G:G |
| 5 | RathuHeenati | T:T | A:A | G:G | C:C | A:A |
| 6 | Babawee | T:T | A:A | G:G | ? | A:A |
| 7 | ARC 10550 | C:C | A:A | G:G | C:C | A:G |
| 8 | Swarnalatha | C:C | A:A | G:G | ? | A:G |
| 9 | T12 | T:T | C:C | A:G | C:C | G:G |
| 10 | Chinsaba | T:T | A:A | G:G | ? | A:G |
| 11 | Pokkali | C:C | C:C | G:G | C:C | G:G |
| 12 | IR65482-7-216 | C:C | C:C | G:G | C:C | G:G |
| 13 | IR71033-121-15 | C:C | C:C | A:G | C:C | G:G |
| 14 | MUT NS1 | T:T | A:A | G:G | C:C | A:A |
| 15 | OM 4498 | C:C | C:C | G:G | C:C | G:G |
| 16 | RP2068-18-3-5 | C:C | C:C | G:G | C:C | G:G |
| 17 | MO1 | T:T | A:A | G:G | ? | A:A |
| 18 | MTU 1010 | T:T | A:A | G:G | C:C | A:A |
| 19 | RP BIO 4918-230S | T:T | C:C | G:G | C:C | G:G |
| 20 | IR 26 | ? | C:C | A:G | C:C | A:A |
| 21 | IR 40 | C:C | A:A | G:G | ? | A:A |
| 22 | IR 66 | T:T | A:A | G:G | ? | A:A |
| 23 | IR 72 | T:T | A:A | A:G | C:C | G:G |
| 24 | Utrirajappan | T:T | C:C | G:G | C:C | G:G |
| 25 | Ndiang Marie | T:T | A:A | G:G | C:C | A:A |
| 26 | Sinna Sivappu | T:T | A:A | G:G | ? | A:A |
| 27 | Balamwee | T:T | A:A | G:G | C:C | ? |
| 28 | IR 62 | T:T | A:A | A:G | C:C | G:G |
| 29 | RathuHeenati accession | T:T | A:A | G:G | C:C | G:G |
| 30 | IR65482-136-2-2 | T:T | A:A | A:G | C:C | G:G |
| 31 | M1 | T:T | A:A | G:G | C:C | A:A |
| 32 | M4 | T:T | C:C | A:A | C:C | G:G |
| 33 | M61 | T:T | A:A | A:A | C:C | G:G |
| 34 | M80 | T:T | C:C | A:A | C:C | G:G |
| 35 | M88 | T:T | A:A | G:G | C:C | A:A |
| 36 | M123 | T:T | C:C | G:G | C:C | A:A |
| 37 | M131 | T:T | A:A | G:G | C:C | A:A |
| 38 | M179 | T:T | A:A | G:G | C:C | A:A |
| 39 | M182 | C:C | A:A | G:G | C:C | A:A |
| 40 | M187 | C:C | C:C | A:A | C:C | A:A |
| 41 | M189 | C:C | C:A | ? | ? | A:A |
| 42 | M190 | C:C | C:A | ? | ? | A:A |
| 43 | M192 | T:T | C:C | A:G | C:C | G:G |
| 44 | M201 | T:T | A:A | G:G | C:C | A:A |
| 45 | M227 | C:C | C:C | G:G | C:C | G:G |

| **S.No.** | **Genotype** | **snpOS00922** | **snpOS00923** | **snpOS00924** | **snpOS00925** | **snpOS00926** |
| --- | --- | --- | --- | --- | --- | --- |
| 46 | M240 | T:T | A:A | G:G | C:C | A:A |
| 47 | M262 | C:C | A:A | G:G | C:C | A:A |
| 48 | M267 | T:T | A:A | G:G | C:C | A:A |
| 49 | M272 | T:T | A:A | G:G | C:C | A:A |
| 50 | M276 | T:T | C:C | G:G | C:C | A:A |
| 51 | M278 | C:C | C:C | A:G | C:C | G:G |
| 52 | M279 | T:T | A:A | G:G | C:C | A:A |
| 53 | M284 | T:T | A:A | G:G | C:C | A:A |
| 54 | M286 | T:T | C:A | G:G | C:C | A:G |
| 55 | M289 | C:C | C:C | A:G | C:C | G:G |
| 56 | M293 | T:T | A:A | G:G | C:C | A:A |
| 57 | M296 | C:C | C:C | G:G | C:C | G:G |
| 58 | M304 | C:T | C:A | G:G | C:C | A:G |
| 59 | M306 | T:T | A:A | G:G | C:C | A:A |
| 60 | M312 | C:C | C:C | A:G | C:C | G:G |
| 61 | M359 | C:C | C:C | A:A | C:C | G:G |
| 62 | M344 | T:T | A:A | G:G | C:C | A:A |
| 63 | M362 | T:T | A:A | G:G | C:C | A:A |
| 64 | M364 | C:C | A:A | G:G | C:C | A:A |
| 65 | M384 | T:T | A:A | G:G | C:C | A:A |
| 66 | IET23993 | T:T | A:A | G:G | C:C | G:G |
| 67 | BM71 | T:T | C:C | G:G | C:C | A:A |
| 68 | RPV1355 | T:T | A:A | G:G | C:C | G:G |
| 69 | KNM118 | T:T | A:A | G:G | C:C | A:A |
| 70 | 10-3 | T:T | C:C | A:A | C:C | G:G |
| 71 | Telangana Sona | C:C | A:A | G:G | C:C | G:G |
| 72 | M229 | T:T | C:C | G:G | C:C | G:G |
| 73 | 3K-19 | T:T | A:A | G:G | C:C | A:A |
| 74 | 3K-47 | C:C | C:C | G:G | C:C | G:G |
| 75 | 3K-53 | C:C | C:C | G:G | C:C | A:A |
| 76 | 3K-59 | T:T | A:A | G:G | ? | A:A |
| 77 | 3K-132 | T:T | A:A | G:G | C:C | G:G |
| 78 | 3K-168 | T:T | A:A | G:G | C:C | A:A |
| 79 | 3K-187 | T:T | A:A | G:G | ? | A:A |
| 80 | 3K-200 | T:T | A:A | G:G | C:C | A:G |
| 81 | 3K-202 | T:T | A:A | G:G | C:C | G:G |
| 82 | 3K-290 | T:T | A:A | G:G | ? | A:G |
| 83 | 3K-322 | C:C | A:A | G:G | C:C | G:G |
| 84 | PTB33 | T:T | A:A | G:G | ? | G:G |
| 85 | RathuHeenati | T:T | A:A | G:G | C:C | A:A |
| 86 | RP2068-18-3-5 | C:C | C:C | G:G | C:C | G:G |
| 87 | TN1 | C:C | C:C | ? | ? | A:A |
| 88 | BPT5204 | T:T | C:C | G:G | C:C | G:G |

| **S.No.** | **Genotype** | **snpOS00927** | **snpOS00928** | **snpOS00929** | **snpOS00930** | **snpOS00931** |
| --- | --- | --- | --- | --- | --- | --- |
| 1 | Mudgo | A:A | G:G | G:G | G:G | C:C |
| 2 | IR 64 | T:A | A:G | G:G | A:G | C:C |
| 3 | ASD 7 | A:A | G:G | G:G | G:G | C:C |
| 4 | Milyang 63 | A:A | G:G | G:G | G:G | C:C |
| 5 | RathuHeenati | T:T | G:G | A:A | G:G | C:C |
| 6 | Babawee | T:T | G:G | A:A | G:G | C:C |
| 7 | ARC 10550 | T:T | G:G | A:A | G:G | C:C |
| 8 | Swarnalatha | T:T | G:G | G:A | G:G | C:C |
| 9 | T12 | T:T | G:G | G:A | G:G | C:C |
| 10 | Chinsaba | T:T | G:G | A:A | G:G | C:C |
| 11 | Pokkali | A:A | G:G | G:G | G:G | C:C |
| 12 | IR65482-7-216 | A:A | G:G | G:G | G:G | C:C |
| 13 | IR71033-121-15 | A:A | G:G | G:G | G:G | C:C |
| 14 | MUT NS1 | T:T | A:A | G:A | A:A | A:A |
| 15 | OM 4498 | A:A | G:G | G:G | G:G | C:C |
| 16 | RP2068-18-3-5 | A:A | G:G | G:G | G:G | C:C |
| 17 | MO1 | T:T | G:G | A:A | G:G | C:C |
| 18 | MTU 1010 | T:T | A:A | A:A | A:A | C:C |
| 19 | RP BIO 4918-230S | A:A | G:G | G:G | G:G | C:C |
| 20 | IR 26 | ? | G:G | G:G | G:G | C:C |
| 21 | IR 40 | T:T | G:G | A:A | G:G | C:C |
| 22 | IR 66 | T:T | G:G | A:A | G:G | C:C |
| 23 | IR 72 | A:A | G:G | G:G | G:G | C:C |
| 24 | Utrirajappan | A:A | G:G | G:G | G:G | C:C |
| 25 | Ndiang Marie | T:T | G:G | G:A | G:G | C:C |
| 26 | Sinna Sivappu | T:T | G:G | A:A | G:G | C:C |
| 27 | Balamwee | T:T | G:G | G:A | G:G | C:C |
| 28 | IR 62 | A:A | G:G | G:G | G:G | C:C |
| 29 | RathuHeenati accession | T:T | G:G | A:A | A:A | C:C |
| 30 | IR65482-136-2-2 | A:A | G:G | G:G | G:G | C:C |
| 31 | M1 | T:T | A:A | G:A | A:A | A:A |
| 32 | M4 | A:A | G:G | G:G | G:G | C:C |
| 33 | M61 | A:A | G:G | G:G | G:G | C:C |
| 34 | M80 | A:A | G:G | G:G | G:G | A:A |
| 35 | M88 | T:T | A:A | G:A | A:A | C:C |
| 36 | M123 | T:T | A:A | A:A | A:A | A:A |
| 37 | M131 | T:T | A:A | G:A | A:A | A:A |
| 38 | M179 | T:T | A:A | G:A | A:A | C:C |
| 39 | M182 | T:T | A:A | G:G | A:A | C:C |
| 40 | M187 | A:A | G:G | G:G | G:G | C:C |
| 41 | M189 | T:T | G:G | G:A | ? | C:C |
| 42 | M190 | ? | A:A | A:A | A:A | C:C |
| 43 | M192 | A:A | G:G | G:G | G:G | C:C |
| 44 | M201 | T:T | A:A | G:A | A:A | C:C |
| 45 | M227 | A:A | G:G | G:G | G:G | C:C |

| **S.No.** | **Genotype** | **snpOS00927** | **snpOS00928** | **snpOS00929** | **snpOS00930** | **snpOS00931** |
| --- | --- | --- | --- | --- | --- | --- |
| 46 | M240 | T:T | A:A | A:A | A:A | A:A |
| 47 | M262 | T:T | A:A | A:A | A:A | A:A |
| 48 | M267 | T:T | A:A | G:A | A:A | A:A |
| 49 | M272 | T:T | A:A | A:A | A:A | C:C |
| 50 | M276 | T:T | A:A | A:A | A:A | C:C |
| 51 | M278 | A:A | G:G | G:G | G:G | C:C |
| 52 | M279 | T:T | A:A | A:A | A:A | C:C |
| 53 | M284 | T:T | A:A | A:A | A:A | ? |
| 54 | M286 | T:A | A:G | G:G | A:G | C:C |
| 55 | M289 | A:A | G:G | G:G | G:G | C:C |
| 56 | M293 | T:T | A:A | A:A | A:A | A:A |
| 57 | M296 | A:A | G:G | G:G | G:G | C:C |
| 58 | M304 | T:A | A:G | G:G | A:G | C:C |
| 59 | M306 | T:T | A:A | G:A | A:A | C:C |
| 60 | M312 | A:A | G:G | G:G | G:G | A:A |
| 61 | M359 | A:A | G:G | G:G | G:G | C:C |
| 62 | M344 | T:T | A:G | A:A | A:G | C:C |
| 63 | M362 | T:T | A:A | A:A | A:A | C:C |
| 64 | M364 | T:T | A:A | A:A | A:A | C:C |
| 65 | M384 | T:T | A:A | G:A | A:A | C:C |
| 66 | IET23993 | A:A | G:G | G:G | G:G | C:C |
| 67 | BM71 | T:T | A:A | G:A | A:A | C:C |
| 68 | RPV1355 | A:A | G:G | G:G | G:G | C:C |
| 69 | KNM118 | T:T | A:A | Uncallable | A:A | C:C |
| 70 | 10-3 | A:A | G:G | G:G | G:G | C:C |
| 71 | Telangana Sona | A:A | G:G | G:G | G:G | C:C |
| 72 | M229 | A:A | G:G | G:G | G:G | C:C |
| 73 | 3K-19 | T:T | G:G | G:A | G:G | A:A |
| 74 | 3K-47 | T:A | G:G | G:G | G:G | C:C |
| 75 | 3K-53 | T:T | G:G | A:A | G:G | A:A |
| 76 | 3K-59 | T:T | G:G | A:A | G:G | C:C |
| 77 | 3K-132 | A:A | G:G | G:G | G:G | C:C |
| 78 | 3K-168 | T:T | G:G | A:A | G:G | C:C |
| 79 | 3K-187 | T:T | G:G | G:A | G:G | C:C |
| 80 | 3K-200 | T:T | G:G | A:A | G:G | A:A |
| 81 | 3K-202 | A:A | G:G | G:G | G:G | C:C |
| 82 | 3K-290 | T:T | G:G | G:A | G:G | C:C |
| 83 | 3K-322 | T:T | G:G | G:A | A:A | C:C |
| 84 | PTB33 | T:T | G:G | A:A | G:G | C:C |
| 85 | RathuHeenati | T:T | G:G | A:A | G:G | C:C |
| 86 | RP2068-18-3-5 | A:A | G:G | G:G | G:G | C:C |
| 87 | TN1 | A:A | ? | G:G | G:G | ? |
| 88 | BPT5204 | A:A | G:G | G:G | G:G | C:C |

# Supplementary Table S6. Genotypes with confirmed *Bph17* specific SNPs

| **S.No.** | **Genotypes possessing favourable allele** | **SNPs and their favourable alleles specific to *Bph17*** | | |
| --- | --- | --- | --- | --- |
|  |  | **snpOS00429**  **T:T** | **snpOS00430**  **G:G** | **snpOS00431**  **G:G** |
|  | **MAGIC Lines** | | | |
| 1 | M1 | T:C | G:A | C:G |
| 2 | M4 | C:C | A:A | G:G |
| 3 | M179 | T:T | G:G | G:G |
| 4 | M201 | T:T | G:G | G:G |
| 5 | M262 | T:C | G:A | C:G |
| 6 | M272 | T:T | G:G | G:G |
| 7 | M286 | T:C | G:A | C:G |
| 8 | M306 | T:T | G:G | G:G |
| 9 | M344 | T:T | G:G | G:G |
|  | **Gene Differentials** | | | |
|  | RathuHeenati | T:T | G:G | G:G |
| 10 | Babawee | T:T | G:G | G:G |
| 11 | RathuHeenati accession | T:T | G:G | G:G |
| 12 | IR72 | T:T | G:G | G:G |
| 13 | IR40 | T:T | G:G | C:G |
| 14 | IR 65482-7-216 | T:C | G:A | NA |
|  | **3K- Genome Lines** | | | |
| 15 | 3K-290 | T:C | G:A | C:G |

**Supplementary Table S7. Genotypes with confirmed *Bph32* specific SNPs**

| **S.No.** | **Genotypes** | **Allele detected** |
| --- | --- | --- |
|  | **MAGIC Lines** | |
| 1 | M1 | G:C |
| 2 | M4 | G:C |
| 3 | M80 | G:G |
| 4 | M88 | G:G |
| 5 | M201 | G:G |
| 6 | M240 | G:C |
| 7 | M192 | G:G |
| 8 | M182 | G:G |
| 9 | M187 | G:G |
| 10 | M267 | G:G |
| 11 | M272 | G:G |
| 12 | M276 | G:C |
| 13 | M278 | G:G |
| 14 | M279 | G:G |
| 15 | M284 | G:G |
| 16 | M286 | G:C |
| 17 | M293 | G:G |
| 18 | M296 | G:G |
| 19 | M304 | G:G |
| 20 | M344 | G:G |
| 21 | M359 | G:C |
| 22 | M364 | G:G |
| 23 | M384 | G:G |
|  | **Gene differentials** | |
| 24 | RathuHeenati | G:G |
| 25 | RathuHeenati accession | G:G |
| 26 | IR62 | G:G |
| 27 | Balamwee | G:G |
| 28 | IR66 | G:G |
| 29 | IR64 | G:C |
| 30 | MTU1010 | G:G |
| 31 | Mudgo | G:G |
|  | **Lines** | |
| 32 | PTB33 | G:G |
| 33 | KNM118 | G:G |
| 34 | RDR1200 | G:G |
| 35 | RPV1355 | G:G |
| 36 | BM71 | G:G |
|  | **3K Genome Lines** | |
| 37 | 3K-59 | G:G |
| 38 | 3K-53 | G:G |
| 39 | 3K-47 | G:G |
| 40 | 3K-322 | G:C |
| 41 | 3K-202 | G:G |
| 42 | 3K-200 | G:G |
| 43 | 3K-19 | G:G |
| 44 | 3K-187 | G:G |
| 45 | 3K-168 | G:G |
| 46 | 3K-132 | G:G |

**Supplementary Table S8. Alleles detected for functional SNP related to *Bph9* validated in the study**

| **Lines** | **Allele detected**  **(Favourable allele A:A)** |
| --- | --- |
| Pokkali (donor) | C:C |
| Chinsaba | A:A |
| MO1 | A:A |
| Sinna Sivappu | A:A |
| M278 | A:A |
| M229 | A:C |
| M276 | A:C |
